# Protein sumoylation and phosphorylation intersect in Arabidopsis signaling

**DOI:** 10.1111/tpj.13575

**Published:** 2017-06-04

**Authors:** Ella Nukarinen, Konstantin Tomanov, Ionida Ziba, Wolfram Weckwerth, Andreas Bachmair

**Affiliations:** ^1^ Department of Ecogenomics and Systems Biology BZA University of Vienna Vienna Austria; ^2^ Department of Biochemistry and Cell Biology Center for Molecular Biology Max F. Perutz Laboratories Vienna Austria; ^3^ Vienna Metabolomics Center University of Vienna A‐1060 Vienna Austria

**Keywords:** SUMO, sumoylation, protein phosphorylation, proteome analysis, phosphoproteome, stress response, SIZ1, PIAL, *Arabidopsis thaliana*

## Abstract

Conjugation of the small ubiquitin‐related modifier (SUMO) to protein substrates has an impact on stress responses and on development. We analyzed the proteome and phosphoproteome of mutants in this pathway. The mutants chosen had defects in SUMO ligase SIZ1, which catalyzes attachment of single SUMO moieties onto substrates, and in ligases PIAL1 and PIAL2, which are known to form SUMO chains. A total of 2657 proteins and 550 phosphopeptides were identified and quantified. Approximately 40% of the proteins and 20% of the phosphopeptides showed differences in abundance in at least one of the analyzed genotypes, demonstrating the influence of SUMO conjugation on protein abundance and phosphorylation. The data show that PIAL1 and PIAL2 are integral parts of the SUMO conjugation system with an impact on stress response, and confirm the involvement of SIZ1 in plant defense. We find a high abundance of predicted SUMO attachment sites in phosphoproteins (70% versus 40% in the total proteome), suggesting convergence of phosphorylation and sumoylation signals onto a set of common targets.

## Significance Statement

The proteome of mutants in SUMO conjugation displays abundant changes in metabolic and regulatory pathway components, in particular those related to stress response. Phospho‐proteome analysis indicates that sumoylation and phosphorylation share a set of common targets, which may thereby integrate stimuli from different signaling cascades.

## Introduction

Post‐translational modification (PTM) of proteins can cause changes in stability, activity, interaction or subcellular localization. One and the same protein can be modified by different PTMs, either at the same time or at different time points in their life cycle, or depending on external cues. Protein phosphorylation is one of the most common PTMs in eukaryotic cells. Its reversible and highly dynamic occurrence is coordinated by protein kinases and phosphatases. Another PTM is sumoylation, the covalent attachment of small ubiquitin‐related modifier (SUMO) protein to lysine (Lys) residues of target proteins (Novatchkova *et al*., 2004, 2012; Castro *et al*., 2012; Xu and Yang, 2013; Elrouby, 2015). Sumoylation is mediated by the heterodimeric SUMO‐activating enzyme (subunits SAE1 and SAE2 in Arabidopsis), by SUMO‐conjugating enzyme (SCE1 in Arabidopsis) and by SUMO E3 and E4 ligases (SIZ1, HPY2, PIAL1 and PIAL2 in Arabidopsis) (Miura *et al*., 2005; Huang *et al*., 2009; Ishida *et al*., 2009; Tomanov *et al*., 2014). SUMO‐activating enzyme first forms a thioester with the carboxyl terminus of SUMO. Activated SUMO is then transferred to SUMO‐conjugating enzyme SCE, which can link SUMO to ε‐amino groups of Lys residues in the substrate. In many cases, SUMO ligases increase the specificity of substrate selection and/or the rate of modification. A typical sumoylation substrate carries a single SUMO moiety, but chains of SUMOs have also been observed.

Mutants in SUMO conjugation have made a significant contribution to functional analysis of sumoylation. The *siz1* mutant shows defects in survival of cold, heat and other stresses (for a review, see Castro *et al*., 2012; Xu and Yang, 2013). In addition, it shows a severe reduction of growth and changes in plant architecture. Much of the growth defect has been linked to the constitutively high levels of salicylic acid (SA), because mutations in SA synthesis, or increased SA turnover, largely reverse the reduced growth phenotype (Lee *et al*., 2007). In contrast, inactivating the two homologous SUMO ligases PIAL1 and PIAL2 maintains wild‐type (WT) growth characteristics. However, mutants show increased salt resistance and decreased resistance to osmotic stress (Tomanov *et al*., 2014). The growth habit of *pial1 pial2 siz1* triple mutants is generally similar to the *siz1* single mutant. Numerous *in vivo* substrates have been identified for SUMO ligase SIZ1 (e.g. Miura *et al*., 2007; Park *et al*., 2011a), but none so far for PIAL1/2. For this reason, it is currently unclear how PIAL1/2 are integrated into cellular sumoylation processes. PIAL1/2 have been analyzed for their ability to form SUMO chains, but recent work also showed a function in transcriptional repression of transposable elements, in a complex that contains the MOM1 protein (Han *et al*., 2016). One of the goals of this work was to better understand how PIAL1/2 intersect with SIZ1, a SUMO ligase with mono‐sumoylation activity. *pial1 pial2* mutants were compared with the Col‐0 WT by quantitative proteomics, as well with to the *siz1* mutant and the triple mutant *pial1 pial2 siz1*. Analysis of differences in protein abundance indicates that the functions of SIZ1 and PIAL1/2 are interdigitated to a surprising extent.

Combinatorial PTMs play an important role in many cellular events (Seet *et al*., 2006; Hendriks *et al*., 2014). A connection between SUMO conjugation and ubiquitylation (the covalent attachment of small modifier ubiquitin to target proteins) was revealed by characterization of ubiquitin ligases with specificity for multiply sumoylated substrates (Sriramachandran and Dohmen, 2014). Most of the substrates for this sumoylation–ubiquitylation cascade may actually carry SUMO chains, as formed by Arabidopsis SUMO ligases PIAL1/2 (Tomanov *et al*., 2014), or by the mammalian open reading frame (ORF) ZNF451 (Cappadocia *et al*., 2015; Eisenhardt *et al*., 2015). One consequence of this pathway is that SUMO conjugation impinges on protein stability for a selected subset of sumoylation substrates. SUMO conjugation is also intertwined with phosphorylation in several ways. For instance, sumoylation coincides with activation and turnover of sucrose non‐fermenting related protein kinase 1 (SnRK1) (Crozet *et al*., 2016), and phosphorylation and sumoylation both affect the same transcription factor, CESTA (Khan *et al*., 2014). Also, nitrate reductase activity is antagonistically regulated by sumoylation and phosphorylation (Park *et al*., 2011a).

Quantitative phosphoproteomics, as a tool to capture the dynamics and specificity of protein phosphorylation, can be used to compare different physiological and developmental states (for recent reviews, see Li *et al*., 2015; Silva‐Sanchez *et al*., 2015). Mutants in protein kinase activity have also been employed (Nukarinen *et al*., 2016). In this work, we extended this principle to mutants in sumoylation, a pathway that is a priori unrelated to phosphorylation. Deviations from WT in phosphoprotein content would be indicative of links between the two pathways. As mentioned above, there are several examples of intersection between sumoylation and phosphorylation, but the general scope of such connections is unclear at the moment. This work reveals numerous phosphorylation events that are influenced by sumoylation, and shows that sumoylation and phosphorylation have a predicted set of common targets, which may be points of convergence for two distinct signaling paths. Finally, the work provides a resource data set of proteins with an abundance that is, directly or indirectly, affected by sumoylation.

## Results

### Proteome analysis of Col‐0 in comparison with sumoylation mutants

We analyzed the proteome and phosphoproteome by an LC‐MS shotgun technique. Proteins were extracted from plant samples comprising the whole aerial part of Col‐0 WT, *pial1 pial2*,* siz1* and *pial1 pial2 siz1* mutants. From total proteomics data, the proteins that were identified and quantified in all five biological replicates in at least one genotype were included into the final data matrix (Table S1). This data matrix contains 2657 proteins. The data were further filtered so that only proteins for which the abundance was significantly (anova *P *<* *0.05), and at least 50%, different between any two genotypes, were kept for further analyses (1034 proteins; Table S1 in the Supporting Information). To generate the phosphopeptide data matrix, the identified and quantified phosphopeptides were filtered as the total protein data set. In addition, only peptides that had a phosphorylation localization probability >0.75 and score difference >5 were kept. After filtering, a data matrix of 550 phosphopeptides (Table S2) was obtained. Generally, the pairwise differences Col‐0 WT versus *pial1 pial2* and *siz1* versus *pial1 pial2 siz1* seem particularly informative, because of the similar growth habit of these pairs. The differences were smallest in the Col‐0 versus *pial1 pial2* comparison. In contrast, and as expected from previous analyses and from growth characteristics, a Col‐0 WT versus *siz1* comparison shows extensive changes in the mutant.

### SUMO and SUMO pathway proteins

**Figure 1:**
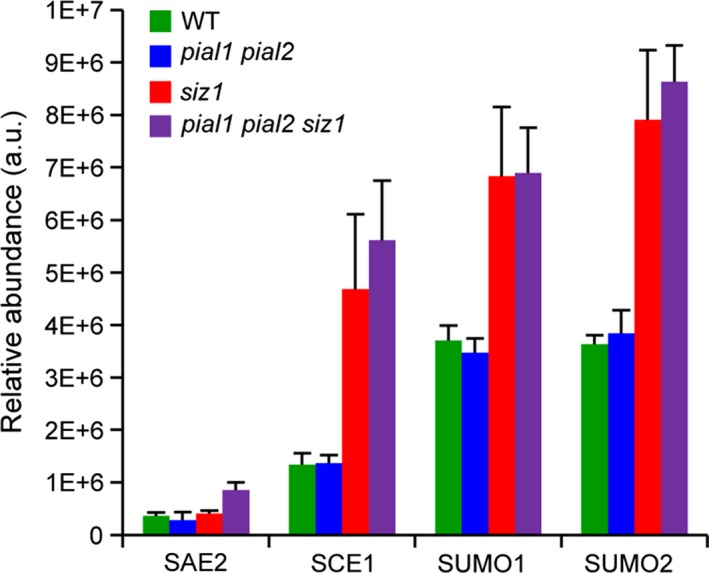
Relative abundance of small ubiquitin‐related modifier (SUMO) and of SUMO pathway proteins in different genotypes. Values represent means of five biological replicates and standard deviation.

With the untargeted total proteomics analysis we were able to detect and quantify SUMO‐activating enzyme 2 (SAE2; AT2G21470), SUMO‐conjugating enzyme 1 (SCE1; AT3G57870) and small ubiquitin‐related modifiers 1 and 2 (SUMO1; AT4G26840 and SUMO2; AT5G55160) (Figure 1). The quantities of SAE2, SCE1, SUMO1 and SUMO2 in the *pial1 pial2* double mutant were at the same level as in the WT, but significantly increased in the mutants lacking SIZ1 (Figure 1). Phosphorylation of SUMO proteins was also detected (Figure S1). Both SUMO1 and SUMO2 are phosphorylated on Ser2. Unlike phosphorylation of SUMO2 on Ser2 and Thr4, which was found in one previous study (Roitinger *et al*., 2015), the phosphorylation of SUMO1 is not currently listed in the PhosPhat 4.0 database or the Plant Protein Phosphorylation DataBase (P3DB). While the level of SUMO is altered by the *siz1* mutation, there is no indication that the phosphorylated fraction of these proteins changes with genotype.

### Proteome differences between Col‐0, *pial1 pial2*,* siz1* and *pial1 pial2 siz1* mutants

**Figure 2:**
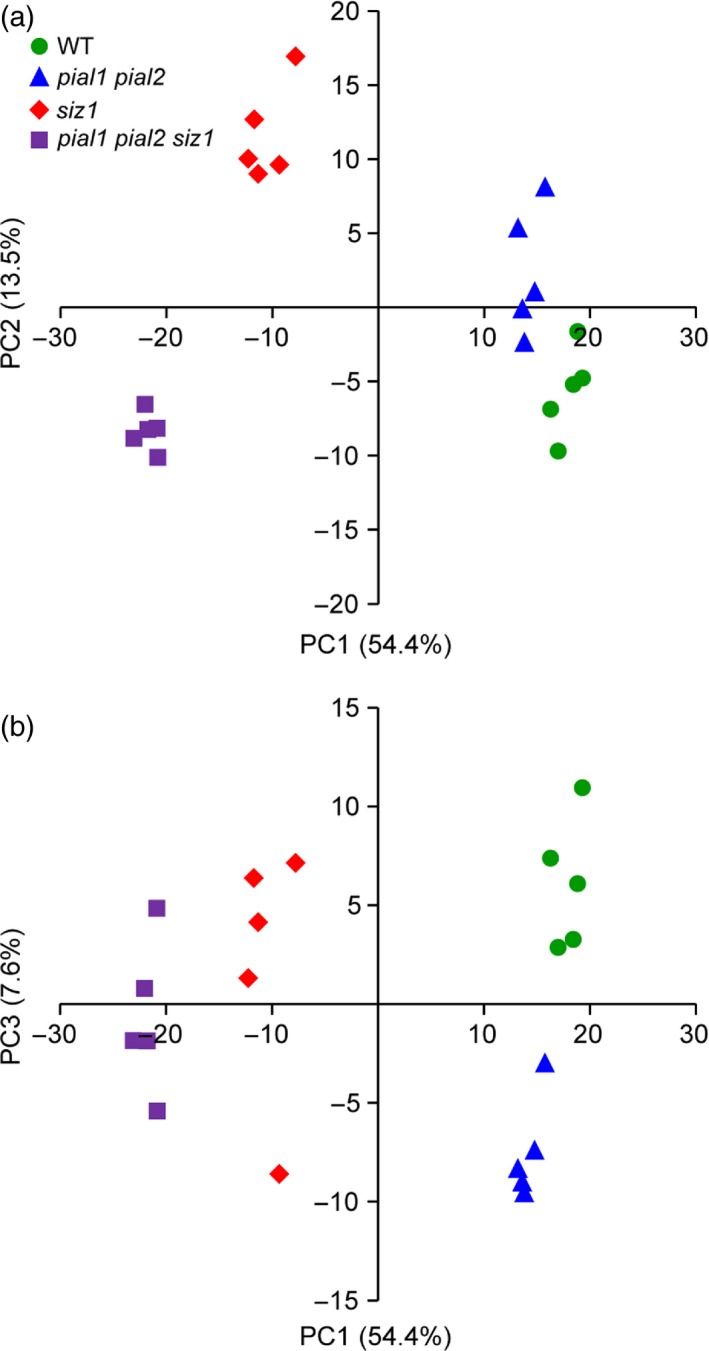
Principal component analysis (PCA) of protein abundance in different genotypes. The PCA includes all significantly changed proteins (anova,* P *<* *0.05) with level differences of at least 50% between any genotype comparisons. Dots are the biological replicates.

**Figure 3:**
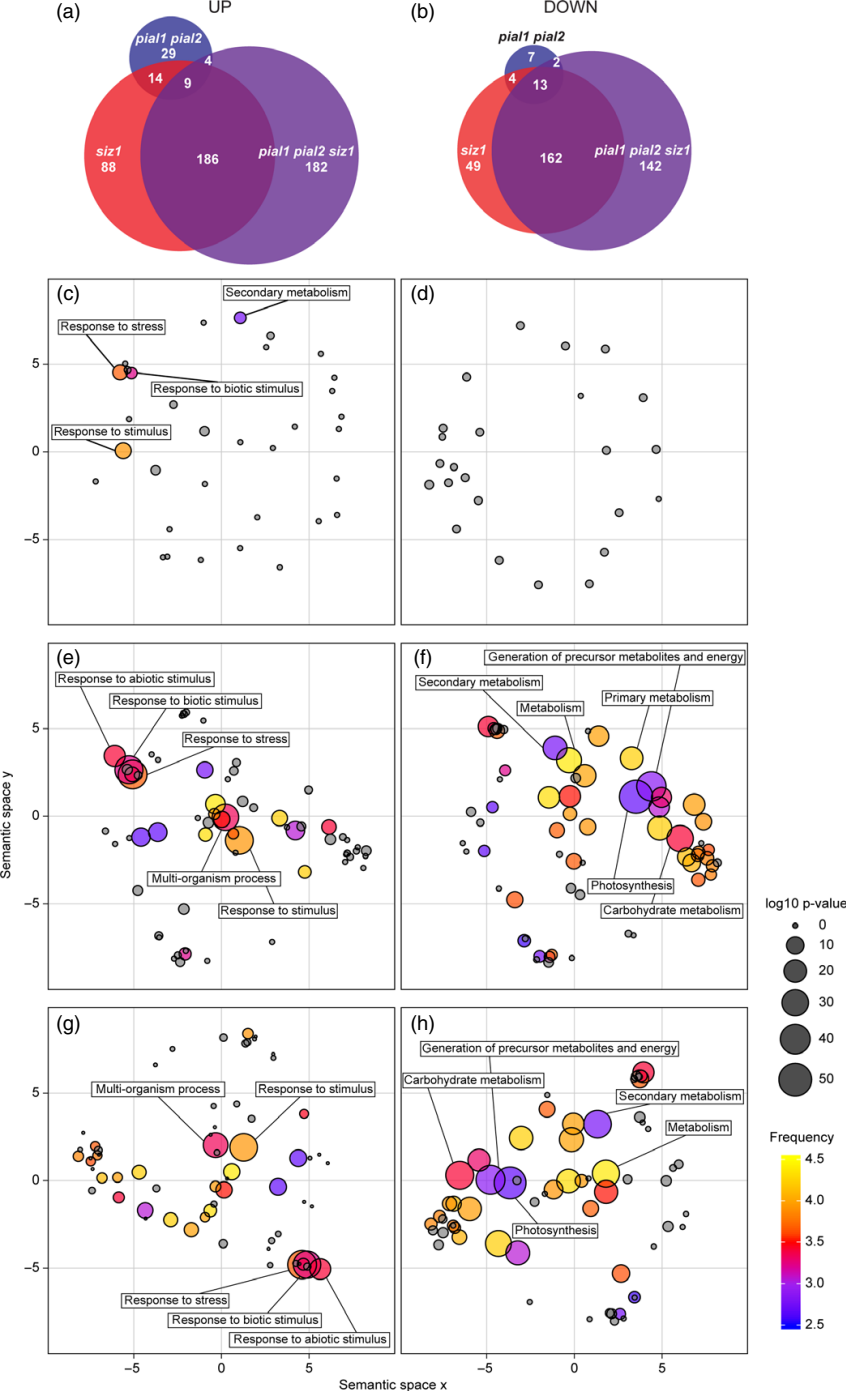
Graphic display of up‐ and downregulated proteins grouped via Gene Ontology (GO) terms. (a) Upregulated and (b) downregulated proteins in different backgrounds in a Venn diagram. Left lower panels, upregulated proteins in *pial1 pial2* (c), *siz1* (e), and in *pial1 pial2 siz1* (g) mutants compared with the wild type. Right lower panels, downregulated proteins in *pial1 pial2* (d), *siz1* (f) and in *pial1 pial2 siz1* (h) mutants compared with the wild type. The analysis includes all significantly changed proteins (anova,* P *<* *0.05) with an abundance change of at least 50% when compared with the wild type. The size of the dots indicates the significance level (false discovery rate). The color code displays the number of Arabidopsis genes summarized under a particular GO term (frequent terms in yellow, rare terms in blue, logarithmic scale). Gray is used for GO terms not enriched above the chosen significance level (*P *>* *0.01). For details, see Experimental Procedures, Figure S2 and Table S4.

Using the data set for WT and the three mutants, protein changes were analyzed via principal component analysis (PCA). Moreover, we did Gene Ontology (GO) annotation with the AgriGO tool and then reduced and compared the semantic similarity of GO terms with REViGO. Consistent with the WT‐like growth, the proteome of the *pial1 pial2* mutant was relatively similar to WT but could still be separated by PCA from the WT (Figure 2; all loading values can be found in Table S3). *pial1 pial2* separated from WT by principal components (PC) 1 and 3, mainly PC3. When the protein abundances were compared between *pial1 pial2* and WT there were more upregulated than downregulated proteins in the mutant (56 upregulated versus 26 downregulated proteins; Figure 3).

Proteins upregulated in the *pial1 pial2* mutant were enriched in GO categories response to stimulus, response to stress and especially biotic stimulus and secondary metabolism (Figure 3c). From the 26 downregulated proteins, six were related to protein synthesis according to MapMan categories, but in the GO analysis no enriched ontologies were found (Figure 3d). Among the 56 upregulated proteins were five 14‐3‐3 proteins (isoforms λ, υ, φ, χ and ω, also known as GRF6, 5, 4, 1 and 2, respectively). 14‐3‐3 proteins operate by binding to phosphoserine or phosphothreonine residues and can dimerize, thereby bringing phosphoproteins into proximity as part of a functional cycle (Paul *et al*., 2009). Five proteins that are part of endoplasmic reticulum (ER) bodies, and glucosinolate metabolism‐related proteins, were also among the proteins with increased abundance. It has been reported that Arabidopsis rosette leaves do not contain constitutive ER bodies, but ER bodies are induced in response to wounding and jasmonic acid (JA). In this study, we analyzed the whole shoot at the early flowering stage of unstressed plants. Identified ER body proteins were PYK10 (AT3G09260), PYK10‐binding protein 1 (PBP1; AT3G16420), Jacalin‐related lectins 33 and 34 (JAL33; AT3G16450 and JAL34; AT3G16460) and GDSL lipase‐like protein 22 (GLL22; AT1G54000). In addition, another Jacalin domain‐containing protein, nitrile specifier protein 1 (NSP1; AT3G16400), which is transcriptionally induced by herbivory and correlates with the amount of nitrile (Burow *et al*., 2009), was found to be more abundant in the *pial1 pial2* mutant than in WT. Likewise, other proteins that are part of glucosinolate metabolism in Brassicaceae and 11 peroxidases were upregulated in the *pial1 pial2* mutant. For additional details on protein changes see Appendix S1 (Supporting Results and Discussion).

Mutants lacking SIZ1 were separated from the WT (and *pial1 pial2* mutants) by PC1 (Figure 2). Differences between *siz1* and the *siz1 pial1 pial2* triple mutant were clearly distinguishable via PC1 and PC2. In the pairwise comparisons WT versus *pial1 pial2* and *siz1* versus *pial1 pial2 siz1*, the latter two genotypes differ more from each other than the former ones. Compared with the WT, 297 and 381 proteins were upregulated in *siz1* and *pial1 pial2 siz1* mutants, respectively (Figure 3a). Significant enrichment of GO was found for proteins that are localized in the cell wall and extracellular region (Table S4) and those involved in cell wall degradation processes (MapMan categorization). Another enriched GO term was ‘stress response’, as summarized in Figure 3(e) and (g) (for the *siz1* mutant and the triple mutant, respectively), and more extensively in Figure S2. SIZ1 plays a role in pathogen defense and the *siz1* mutant has increased SA levels (Lee *et al*., 2007), so that proteins related to biotic stress can be expected to be enriched in the *siz1* background. Consistent with an antagonistic regulation of SA and JA in Arabidopsis (Thaler *et al*., 2012), key enzymes of the JA biosynthetic pathway such as lipoxygenase 2 (LOX2; AT3G45140), allene oxide synthase (AOS; AT5G42650) and allene oxide cyclases 1 and 2 (AOC1; AT3G25760 and AOC2; AT3G25770), are less abundant in the *siz1* and *pial1 pial2 siz1* mutants than in the WT.

In the data set, 228 and 319 proteins in *siz1* and *pial1 pial2 siz1* mutants, respectively, are downregulated compared with the WT (Figure 3b). The major effect is on proteins with a role in photosynthesis and starch metabolism. It has been shown that photosynthesis is impaired in Arabidopsis mutants that are constitutively accumulating SA (Mateo *et al*., 2006), and that photosynthesis is affected by pathogen attack (for review, see Berger *et al*., 2007; Bolton, 2009). Among the 228 proteins with decreased abundance in *siz1*, 64% are localized in the chloroplast according to the AT_CHLORO proteome database (Ferro *et al*., 2010). In addition, the major GO term enriched in the downregulated *siz1* protein set is ‘photosynthesis’ and ‘chloroplast‐related proteins’ (Figure 3f). For downregulated proteins of the *pial1 pial2 siz1* mutant, the major GO term was also ‘photosynthesis’ (Figure 3h), and 57% of these proteins are located in the chloroplast. In contrast, only 8% of the upregulated proteins of *siz1* and in *pial1 pial2 siz1* mutants are in the plastid. For further description see Appendix S1.

The difference in the proteome of *siz1* and *pial1 pial2 siz1* mutants was mainly due to proteins with decreased abundance in the triple mutant compared with the *siz1* mutant. These proteins included several cell wall‐associated proteins such as pectin lyase‐like superfamily proteins and plant invertase/pectin methylesterase inhibitor superfamily proteins, three pollen Ole e1 allergen and extensin family proteins (AT1G29140, AT4G18596 and AT2G16630) and pollen‐specific actin‐binding cytoskeleton regulators profilin 4 and 5 (PRF4; AT4G29340 and PRF5; AT2G19770). The reason for this difference remains to be understood.

### Phosphoproteome profiling of Col‐0, *pial1 pial2*,* siz1* and *pial1 pial2 siz1*

**Figure 4:**
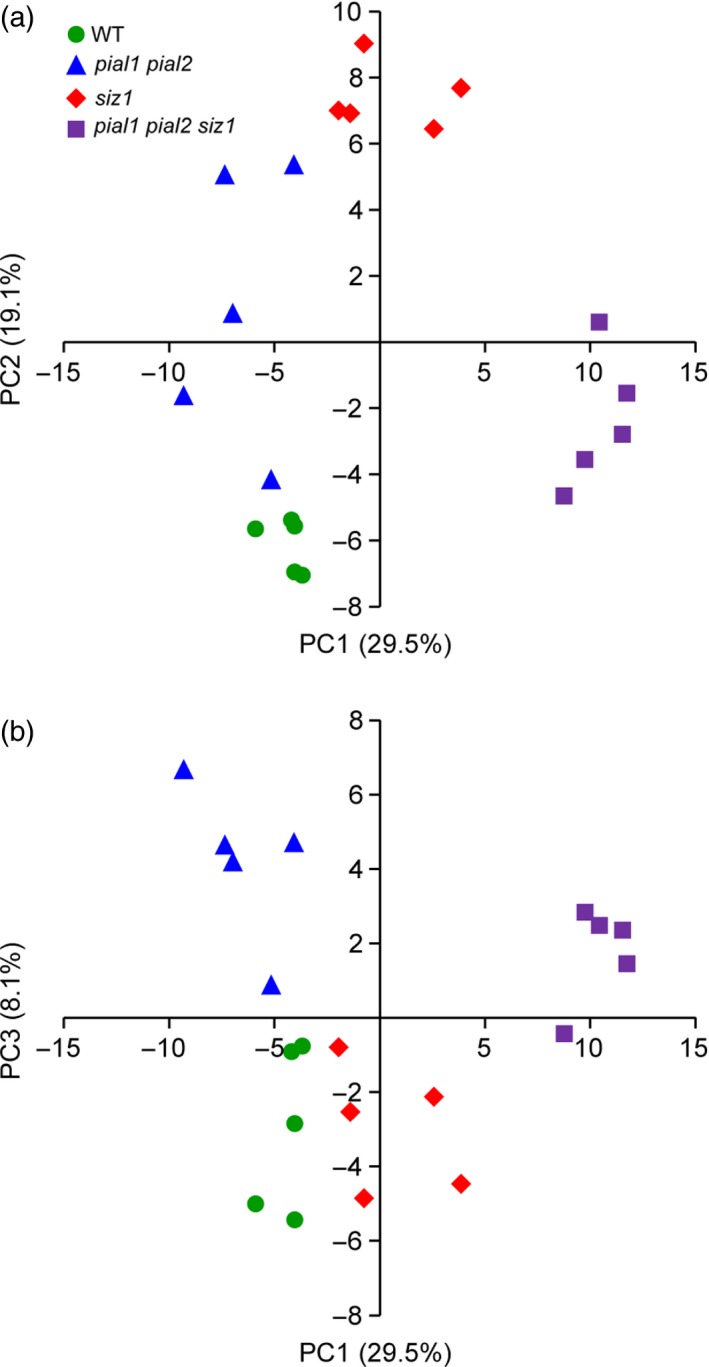
Principal component analysis (PCA) of phosphopeptides. Phosphopeptides that were significantly different (anova,* P *<* *0.05) between at least two genotypes were included in the analysis.

Phosphopeptides that were differently phosphorylated in at least one mutant compared with WT or between mutants were subjected to PCA. The PCA separated mutants from each other and from WT according to the phosphopeptide abundance (Figure 4; the loading values for this PCA are listed in Table S5). This subset of phosphopeptides revealed clearer differences of *pial1 pial2* from the PIAL WT genotypes than analysis of total protein abundance. Genotypes that lack SIZ1 and genotypes that have functional SIZ1 were separated in PC1 (which explains 29.5% of the variance), but PC1 also separated *siz1* from *pial1 pial2 siz1*. PC2 (which explains 19.1% of the variance) separated WT from all the mutant lines, and *siz1* from *pial1 pial2* and *pial1 pial2 siz1*. PC3 (8.1% of the variance) achieved separation of mutants lacking PIAL1 and PIAL2 from genotypes that have functional PIAL1 and PIAL2 proteins.

**Figure 5:**
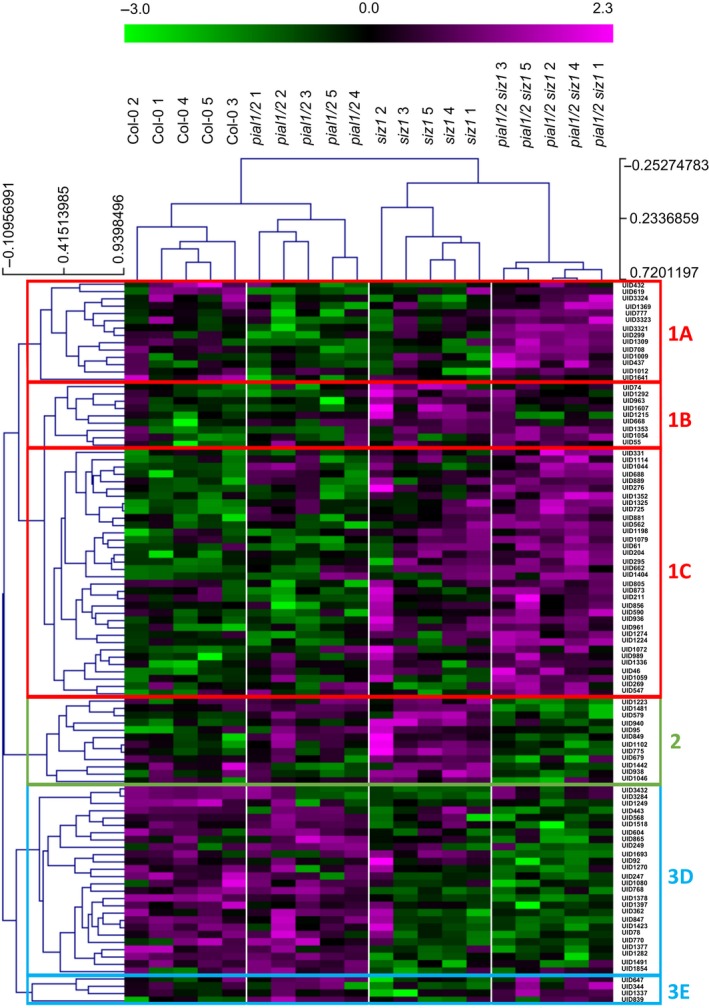
Hierarchical cluster analysis of significantly changed phosphopeptides. All phosphopeptides that were significantly different (anova,* P *<* *0.05) between at least two groups were included in the analysis. Clustering of phosphopeptides and samples was done using Spearman rank correlation with MeV 4.9. Unique identifiers (UID) refer to Table S2.

The phosphopeptide set was also subjected to hierarchical cluster analysis (HCA) (Figure 5). The HCA resulted in three major clusters. The first cluster contains phosphopeptides that were more abundant in the *pial1 pial2 siz1* mutant than in *pial1 pial2* and in the WT. In the second main cluster are phosphopeptides that were differently phosphorylated in *siz1* versus *pial1 pial2 siz1*, with a lower abundance in *pial1 pial2 siz1* than in *siz1*. The third main cluster encompasses proteins with lower abundance in *siz1* than in *pial1 pial2* mutants. At the next hierarchical level, cluster one was further divided into three subclusters: phosphopeptides that are decreased in *pial1 pial2* and increased in the *pial1 pial2 siz1* mutant (subcluster 1A); phosphopeptides with low abundance in WT and in *pial1 pial2*, but high abundance in *siz1* and *pial1 pial2 siz1* (subcluster 1B); finally, other peptides that were more abundantly phosphorylated in the triple mutant (subcluster 1C). Similarly, cluster three can be separated into two subdivisions, one with lower phosphorylation status in *siz1* and *pial1 pial2 siz1* (subcluster 3D) and one with phosphopeptides of higher abundance in the triple mutant than in *siz1* and similar abundance in *pial1 pial2* and the triple mutant (subcluster 3E). For a more detailed description of the clusters and subclusters see Appendix S1. Of note, Figure 5 suggests that, based on phosphoprotein abundances, the difference between *siz1* and *pial1 pial2 siz1* is larger than between Col‐0 and *pial1 pial2*.

**Figure 6:**
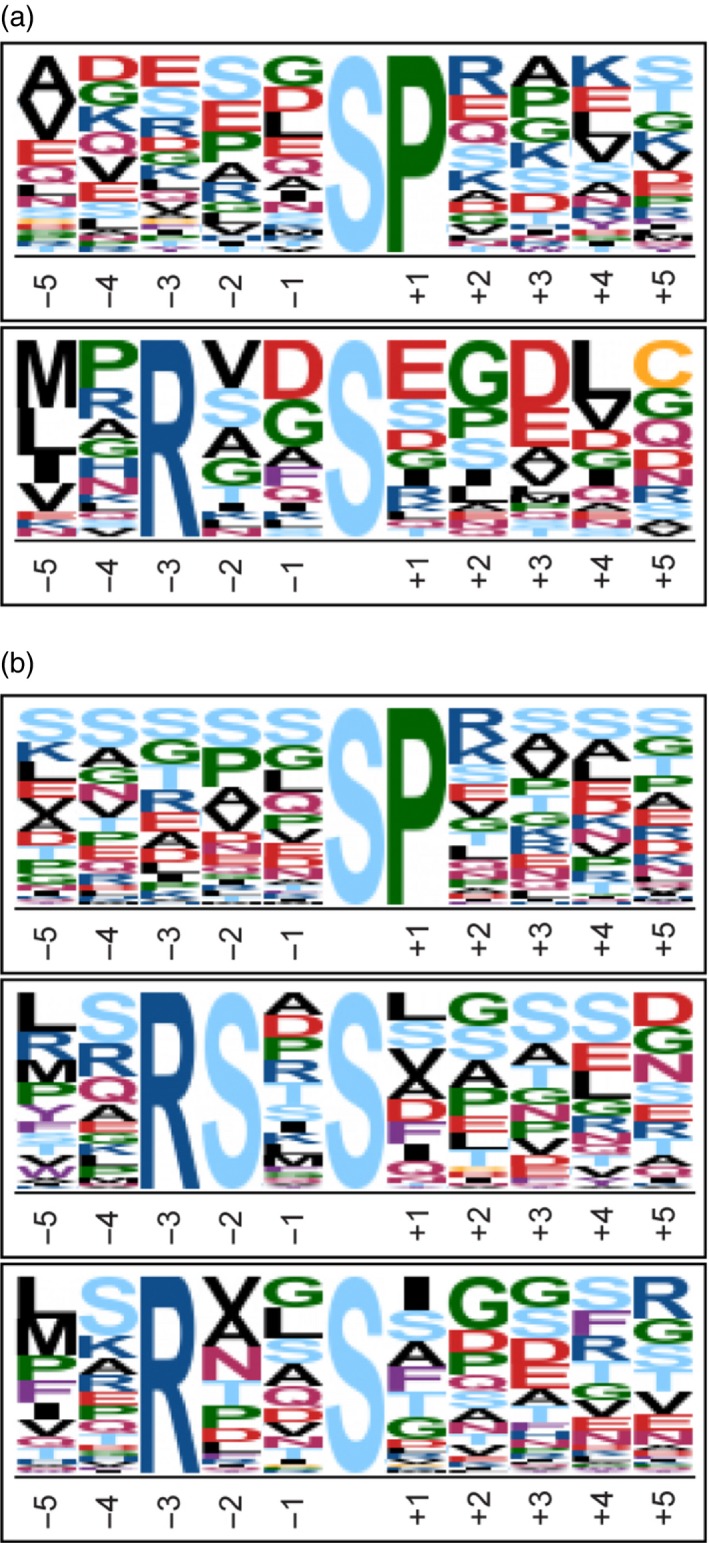
Phosphorylation site composition. Motif enrichment analysis was done with Motif‐x software with settings: >20 occurrences, significance threshold was set to 0.000001 and background proteome was ipi.ARATH database. (a) SP motif score was 16.00 and fold increase 7.09 and R..S motif score was 12.51 and fold increase 7.08. (b) SP motif score was 16.00 and fold increase 7.81, R..S motif score was 16.00 and fold increase 5.29 and RS..S motif score was 27.85 and fold increase 24.46.

Taken together, one conclusion of this study is that sumoylation influences phosphorylation. A follow‐up question is whether proteome data also help to identify critical points of intersection. We searched whether the 54 proteins (represented by 99 phosphopeptides in the data set) with significantly changing phosphorylation status contain known sumoylation motifs. Indeed, several of them contain such motifs. 42 phosphoproteins contain a consensus sumoylation motif, [VIL]K.[DE], 40 contain an inverse consensus motif, [DE].K[VIL], and three of them have a hydrophobic cluster sumoylation motif (HCSM), [VIL][VIL][VIL]K.[DE] (Table 1). As expected from known preferences, the acidic residue [DE] is in most cases Glu [E] (31 of the 42 cases for the consensus motif, 32 of the 40 instances of the inverse consensus motifs). No results within the set of phosphoproteins were found for a negatively charged amino acid‐dependent sumoylation motif (NDSM), [VIL]K.[DE]..[DE][DE][DE][DE], or a phosphorylation‐dependent sumoylation motif (PDSM), [VIL]K.[DE]..SP. The latter motif promotes sumoylation following a prior phosphorylation event in animals (Hietakangas *et al*., 2006; Hendriks *et al*., 2017). Potential SUMO interaction motifs (clusters of four exposed hydrophobic residues followed by S/E/D, and similar motifs; Miteva *et al*., 2010) were identified by pattern search algorithms (GPS‐SUMO 1.0; see Experimental Procedures). Results are shown in Tables 1 and S6, indicating that 122 sumoylation sites were found in 63 of the 90 phosphoproteins (medium cutoff, 81% of the most stringent condition). This 70% probability for the presence of a sumoylation site in the phosphoproteins is an indication that sumoylation and phosphorylation have a significant set of common targets. We also screened the identified phosphopeptides with motif‐x software (http://motif-x.med.harvard.edu/motif-x.html) to search for typical phosphorylation motifs. In the first search, we identified two motifs from the 99 phosphopeptides that were differentially phosphorylated in the genotype comparisons: (i) a typical low‐stringent MAPK motif SP with a fold increase of 7.09 and (ii) a motif with Arg at position −3, and often a hydrophobic residue at position −5 relative to the phosphorylated Ser residue (R..S) with a fold increase of 7.08 (Figure 6a). A second search included those phosphopeptides (451 peptides) that did not change significantly between genotypes. The motifs found in this latter search were similar to the motifs found among significantly changing phosphopeptides (Figure 6b; the SP motif fold increase was 7.81 and the R..S fold increase 5.29). In addition, the larger data set allowed separation of the sequence RS..S as a third motif, with a fold increase of 24.46.

**Table 1:** Summary of sumoylation and phosphorylation motifs in the phosphoproteomics data set. All significantly changed phosphopeptides and corresponding phosphoproteins were included in the analysis

Protein model	Description	Presence of SUMO attachment motifs^a^			Number of SUMO interaction motifs	Phosphorylation motif	
		[VIL]K.[DE]	[DE].K[VIL]	[VIL]3K.[DE]		…SP…	…R..S…
ATCG00480.1	ATP synthase subunit beta		+		2		
AT5G65690.1	PEP carboxykinase 2	+	+		0	+	
AT1G15950.2	Cinnamoyl‐CoA reductase 1		+		3		
AT4G32180.3	Pantothenate kinase 2		+	+	3		+
AT2G46860.1	Pyrophosphorylase 3	+	+		1	+	
AT1G10290.1	Dynamin‐like protein 6	+		+	3		
AT1G59610.1	Dynamin‐like protein 3	+		+	3		
AT3G46780.1	Plastid transcriptionally active 16		+		0		
AT3G13222.1	GBF‐interacting protein 1	+			0	+	
AT1G51140.1	bHLH DNA‐binding protein	+			0		+
AT2G27100.1	C2H2 Zn‐finger protein SERRATE		+		2		
AT5G12850.1	CCCH‐type Zn‐finger protein, ARM repeats	+			1	+	
AT4G28610.1	Phosphate starvation response 1		+		0	+	
AT5G47790.1	SMAD/FHA domain protein	+	+		1	+	
AT3G57540.1	Remorin family protein	+	+		0		
AT3G61260.1	Remorin family protein	+	+		0	+	
AT5G47430.2	CCHC‐type Zn‐finger, DWNN domain		+		1		
AT3G62330.1	CCHC‐type Zn knuckle protein	+	+		0	+	
AT3G51950.2	CCHC‐type Zn‐finger, RRM domain	+			0	+	
AT3G12640.1	RNA binding protein with RRM motifs	+	+		0	+	
AT4G17720.1	RNA binding protein with RRM motifs		+		1	+	
AT5G15270.2	RNA‐binding protein with KH domain	+			1		
AT5G51410.3	LUC7 N‐terminus domain containing	+	+		0	+	
AT1G11480.2	eIF‐related	+	+		1	+	
AT1G69410.1	Eukaryotic elongation factor 5A‐3		+		1		
AT1G51690.2	Protein phosphatase 2A subunit B alpha		+		1		
AT1G79570.1	Protein kinase, Phox domain	+	+		4		
AT5G57610.1	Protein kinase, Phox domain	+	+		1		+
AT3G07610.1	Jumonji (jmjC) domain protein	+	+		3		+
AT4G30890.3	ubiquitin‐specific protease 24	+	+		1		
AT1G76040.1	Ca‐dependent protein kinase 29		+		2		+
AT5G27540.2	MIRO‐related GTPase 1	+			4	+	
AT3G45780.2	Phototropin 1	+	+	+	1	+	
AT4G21450.1	PapD‐like superfamily protein	+			0		
AT1G21390.1	Embryo defective 2170	+	+		0		
AT2G35980.1	Hydroxyproline‐rich glycoprotein family	+			0		
AT5G46750.1	ARF‐GAP domain 9	+	+		1		
AT3G58730.1	V‐ATPase subunit D (VATD)	+			0		+
AT4G13510.1	Ammonium transporter 1;1	+			1	+	
AT1G59870.1	ABC‐type transporter family protein	+	+		1		+
AT5G42950.1	GYF domain protein	+	+	+	1	+	
AT3G13990.2	Kinase‐related protein, DUF1296	+	+	+	2		
AT1G36990.1	Unknown protein	+	+	+	0	+	
AT2G26570.1	Unknown function, DUF827	+	+		0	+	
AT3G05130.1	Unknown protein	+	+		2		
AT3G10650.1	Unknown protein	+	+		0	+	
AT3G46750.1	Unknown protein	+	+		0	+	
AT4G01290.2	Unknown protein	+			1		
AT4G04630.1	Unknown function, DUF584		+		0		
AT4G24680.1	Modifier of snc1	+	+		0		
AT4G32330.3	Targeting protein for Xklp2 family	+	+		0		
AT4G37300.1	Maternal effect embryo arrest 59	+			0		
AT5G20190.1	TPR‐like superfamily protein	+			0		+
AT5G55860.1	Unknown function, DUF827	+	+		1		+

a Listed are data obtained by Protein Pattern Find and GPS‐SUMO 1.0. The amino acid motifs identified are listed in Table S6.

## Discussion

In this work, we compared the proteome and the phosphoproteome of plants with defects in SUMO conjugation with WT plants grown under standard greenhouse conditions. Mutants *siz1*,* pial1 pial2* and the triple mutant *pial1 pial2 siz1* were investigated. PIAL1 and PIAL2 are highly similar with overlapping function and promote SUMO chain formation, whereas SIZ1 is a SUMO ligase that adds single SUMO moieties to its substrates. Whereas loss of SIZ1 causes severe growth reduction and developmental alterations, no impact of the loss PIAL1 and PIAL2 was found on plant growth. Both SIZ1 and PIALs, however, affect stress survival. Because no substrates of PIAL1/2 are currently known, the integration of PIALs into cellular sumoylation events remains to be understood.

One of the questions we asked was whether PIAL1/2 affect the stability of certain proteins, via SUMO chain‐binding ubiquitin ligases (Sriramachandran and Dohmen, 2014). If such a connection exists, loss of PIAL1/2 should result in increased levels of these substrates. Overall statistics give a first hint. A comparison of *pial1 pial2* mutants with the WT shows that 56 of the 2657 proteins surveyed have increased abundance, which amounts to 2% of this sample (Figure 3a,b). In the triple mutant *pial1 pial2 siz1*, 381 proteins have increased abundance compared with the WT (14% of the sample). Candidates for PIAL‐dependent protein turnover might be found in these two sets.

By contrast, only 26 proteins are downregulated in *pial1 pial2* mutants compared with WT. Whereas no GO designation is enriched in the downregulated proteins, the proteins upregulated in *pial1 pial2* are enriched in a specific subset of functions, response to stress and response to stimulus (Figure 3c,d). Comparing the *pial1 pial2* mutation in the *siz1* background (i.e. the triple mutant) with the WT gives 319 downregulated proteins (and 381 upregulated proteins, see above). The *siz1* mutation alone results in 228 downregulated and 297 upregulated proteins (GO terms are listed in Figures 3 and S2). Thus, loss of PIAL1/2 has a greater impact on protein abundances in the *siz1* mutant background than in the WT background. The trend is even more obvious with regard to differences in protein phosphorylation, as revealed by HCA (Figure 5). The increased importance of PIAL function in plants already compromised in sumoylation is indicative of the integration of these E4 ligases into cellular sumoylation activities and stress responses, but the functional connections underlying this result remain to be understood. In the following, we discuss a few of the proteins with changed abundance.

### Upregulation of sumoylation components in mutants

In mutants devoid of SUMO ligase SIZ1 (genotypes *siz1* and *siz1 pial1 pial2*), SUMO1, SUMO2 and SUMO‐conjugating enzyme SCE are upregulated (Figure 1). This result is in agreement with a general homeostasis model in which sub‐optimal sumoylation capacity leads to increased abundance of its components. It has been shown before (Kurepa *et al*., 2003) that SUMO overexpression can increase SUMO conjugation. The cellular upregulation of SCE1 is also plausible in light of the fact that SCE has SUMO ligase activity and can therefore partially substitute for SIZ1. Because the *siz1* and *pial1 pial2 siz1* mutants are very similar in this respect, whereas no effect can be seen in the *pial1 pial2* double mutant, we conclude that this effect can almost exclusively be ascribed to the lack of SIZ1.

### Changes in components of stress response and regulation

This work confirms once more the involvement of SIZ1 in pathogen defense (Castro *et al*., 2012; Elrouby, 2015). Increased abundance of defense‐related compounds may be related to the high level of SA in *siz1* mutants. We find components of glucosinolate biosynthesis upregulated in *siz1* mutants. In line with the fact that Arabidopsis is one of those plants where JA and SA act antagonistically (Thaler *et al*., 2012), our data confirm that an elevated SA level leads to downregulation of JA‐inducible genes and JA biosynthesis genes. Correspondingly, the GO term ‘response to stress’ is prevalent in the data set of protein changes in *siz1* mutants (Figure 3e). *siz1* mutants (genotypes *siz1* and *pial1 pial2 siz1*) also show a broad decrease in proteins related to photosynthesis and starch metabolism. A decrease in photosynthesis might be an active process to limit carbon for pathogens, and/or to prioritize metabolism in the direction of defense (Berger *et al*., 2007; Bolton, 2009).

In contrast, changes in *pial1 pial2* mutants are more subtle, but the GO term ‘response to stress’ is also enriched among proteins with increased abundance (Figure 3c). A coordinated response can be observed in *pial1 pial2* mutants with regard to components of so‐called ER bodies. ER bodies are structures observed mainly in Brassicales that perform defense‐related functions (Nakano *et al*., 2014). A major protein of ER bodies is PYK10, a β‐glucosidase that hydrolyses glucosinolates to produce defense compounds against herbivory and plant pathogens. ER body proteins are tightly co‐regulated with glucosinolate biosynthesis (Matsushima *et al*., 2003; Nakano *et al*., 2017). Park *et al*. (2011b) identified PYK10 as a SUMO1 target under heat stress in seedlings. It remains to be analyzed whether the impact of PIAL1 and PIAL2 on the abundance of these defense‐related genes is direct or indirect. Interestingly, most of the upregulated proteins are under the control of the transcription factor NAI1 (AtbHLH20, AT2G22770). Summarized under the GO term ‘response to stimulus’ in Figure 3(c), a set of 14‐3‐3 proteins also shows increased abundance. Five of the thirteen members of this gene family are affected. 14‐3‐3 proteins participate in the response to phosphorylation signals. Binding domains for P‐Ser/P‐Thr lead to specific interaction with phosphoproteins (Wilson *et al*., 2016). It will therefore be of particular interest to understand the impact of sumoylation on their abundance and activity.

It should be noted that PIAL1 and PIAL2 have recently been ascribed a function as (alternative) subunits of a gene silencing complex that affects mainly transposable elements (Han *et al*., 2016). Analysis of the transcriptome of *pial1 pial2* mutants as published by Han *et al*. (2016) indicated only five genes upregulated at the transcriptional level among our set of 56 significantly upregulated proteins (AT1G26380, AT2G14610, AT2G20560, AT4G04180, AT5G27850). In the other cases, post‐transcriptional steps such as increased protein stability may be responsible for the upregulation.

### Changes in the phosphoproteome

Another goal of this study pertains to links between SUMO conjugation and phosphorylation. Five hundred and fifty phosphopeptides belonging to 411 proteins were detected and quantified. Ninety‐nine phosphopeptides, listed in Figure 5, displayed different abundance among the genotypes. They belong to the 54 proteins of Tables 1 and S2. Mutations in PIAL1 and PIAL2 changed phosphopeptide abundance only marginally or not at all. As found for protein abundance data, the same mutations have a stronger impact when present in the *siz1* background. For example, in the HCA of Figure 5, cluster 1A contains phosphopeptides that show increased abundance in the triple mutant but not in the single *siz1* mutant or in the *pial1 pial2* double mutant. We take this again as an indication that SUMO chain formation by PIALs is integrated into SIZ1‐dependent sumoylation events in a complementary way that has to be elucidated in future work. Most importantly, changes in phosphorylation in any of the sumoylation mutants imply connection(s) between the two post‐translational modifications.

### Motif search to reveal connections between the pathways

The identification of proteins with a changed phosphorylation state prompted a search for phosphorylation‐dependent sumoylation motifs (PDSMs; Hietakangas *et al*., 2006; Hendriks *et al*., 2017). These motifs have previously been described in animals, where a class of proteins is sumoylated only in dependence of a prior phosphorylation event. The added phosphate group allows binding of the sumoylation enzyme SCE to the substrate. If similar motifs exist in plants we expected to observe increased phosphorylation of PDSM substrates in sumoylation mutants, provided that the modification by phosphate is reversed only after the sumoylation step. However, we found no evidence for regulatory modules in which phosphorylation and sumoylation are coupled in this way. This was surprising, given that both phosphorylation and sumoylation regulate stress responses and developmental transitions. In the subset of the 411 phosphoproteins we also could not find consensus sumoylation motifs that are flanked by acidic residues (negatively charged amino acid‐dependent motifs or NDSMs). This is consistent with the fact that phosphorylation adds negative charges to a stretch of amino acids and may thereby convert a poor sumoylation motif into an NDSM.

The negative search result is not due to a lack of consensus sumoylation motifs, however. In general, 70% of the detected phosphoproteins contain sumoylation sites (medium strength; the occurrence in the total Arabidopsis proteome is about 40%; Elrouby and Coupland, 2010). These data indicate that plants may not use phosphorylation–sumoylation cascades in the manner of PDSM motifs. The increased abundance of predicted sumoylation sites in phosphoproteins, in comparison with the general protein population, however, indicates important overlaps in the substrate space. Both phosphorylation and sumoylation impinge on regulatory processes, and a specific set of proteins are apparently hubs for integration of both phosphorylation and sumoylation signals.

In sum, the SUMO chain‐forming ligases PIAL1/2 are integrated into the cellular sumoylation landscape, as demonstrated by increased changes in mutants with additional SUMO conjugation defect (*siz1*). We find increased presence of sumoylation motifs in phosphorylated proteins, but no phosphorylation‐dependent sumoylation sites. In plants, sumoylation and phosphorylation thus seem to probe distinct environmental signals and developmental cues, but these signals can impinge on the same execution pathways.

## Experimental Procedures

### Plant cultivation and sample harvesting

Plants of genotype WT (Col‐0 accession), *siz1‐2* (SALK_065397) single mutant, *pial1‐1* (SALK_083748) *pial2‐2* (GK_712B09) double mutant, and *pial1‐1 pial2‐2 siz1‐2* triple mutant, called *siz1*,* pial1 pial2* and *pial1 pial2 siz1* in the text, were grown on soil in a growth chamber (16‐h day, 8‐h night, 21°C constant temperature, irradiation intensity 170 μmol m^−2^ sec^−1^ by white fluorescence lamps, 60% relative humidity) and harvested at early flowering stage.

### Protein extraction, phosphopeptide enrichment, LC‐MS analysis

Total protein was extracted from 1 ml of ground plant material as previously described (Colby *et al*., 2011). Plant material was mixed with 5 ml of ice‐cold 10% trichloroacetic acid (TCA) in acetone and incubated in an ultra‐sonication water bath for 10 min. Then plant material was washed with 2 × 5 ml ice‐cold 10% TCA in acetone, 1 × 5 ml ice‐cold 10% TCA in water and 2 × 5 ml ice‐cold 80% acetone. Between the washes, the plant material was collected by centrifugation at 4000 ***g*** for 5 min. After the last centrifugation, the supernatant was carefully discarded and the pellet was air dried. Protein extraction was done with 4 ml of dense SDS‐buffer (0.1 m TRIS‐HCl, pH 8.0/30% sucrose/2% SDS/5% β‐mercaptoethanol). Liquid–liquid extraction, first with 4 ml and then with 3 ml of phenol, was performed. Each extraction was done with 1 min of vortexing and phenol and aqueous phases were separated by centrifugation at 4000 ***g*** at room temperature (23°C) for 20 min. Phenol phases were transferred into new tubes and counter‐extracted with 7 ml of dense SDS buffer without SDS. Finally, the phenol phase was transferred into a new tube and protein was precipitated with 35 ml of ice‐cold 0.1 m ammonium acetate in methanol (MeOH) overnight. Protein was collected by centrifugation at 4000 ***g*** for 10 min and washed twice with ice‐cold 0.1 m ammonium acetate in MeOH and twice with ice‐cold 80% acetone. After discarding the last acetone wash, the protein pellet was air dried.

Protein digestion was done as in Nagler *et al*. (2015). The protein pellet was dissolved in 8 m urea, 0.1 m ammonium bicarbonate (AmBic; Merck, http://www.merckmillipore.com/product/Ammonium-hydrogen-carbonate,MDA_CHEM-101131). The amount of protein was determined with a Bradford assay using BSA as a protein standard. For further sample preparation, 1100 μg of total protein was taken. First, cysteine residues were reduced with 5 mm DTT at 37°C for 45 min, then alkylated with 10 mm iodoacetamide at 23°C for 60 min and finally another portion of 5 mm DTT was added and samples were incubated at 23°C for 15 min to prevent over‐alkylation. Second, protein was pre‐digested with LysC (1:1000 w/w) at 30°C for 5 h. Third, after dilution to a urea concentration of 2 m with 50 mm AmBic/10% acetonitrile (ACN) and addition of 2 mm CaCl_2_, protein was digested with immobilized trypsin (1:36 v/w) and incubated at 37°C for 15 h.

Peptides were desalted with C18 and carbon graphite (CG) solid phase extraction (SPE) materials, as described (Furuhashi *et al*., 2014). For C18 desalting, a SPEC C18 96‐well plate containing 15 mg of SPE material was used. The SPE material was activated by 2 × 400 μl of MeOH and equilibrated with 4 × 400 μl of H_2_O. The sample was loaded and desalted with 4 × 400 μl of H_2_O. The sample flow‐through and first two washes were collected and pooled. Peptides were eluted from C18 SPE by 2 × 200 μl of MeOH. The collected sample flow‐through and washes were acidified by addition of trifluoroacetic acid (TFA) to a concentration of 1% and desalted with carbon graphite (CG) SPE. Then 80 μg of CG SPE was activated with 2 × 600 μl of 1 m ammonium hydroxide (NH_4_OH) and 1 × 600 μl of 100% ACN and then equilibrated with 2 × 600 μl of 1% TFA. The samples were allowed to bind for 10 min onto the CG SPE. Desalting was done by washing with 3 × 600 μl of 1% TFA. Peptides were eluted with 2 × 200 μl of 50% ACN/0.1% formic acid (FA). The eluates from C18 and CG SPEs were pooled, split between two tubes (100 μg for total proteomics analysis and 1000 μg for phosphopeptide enrichment) and dried in a vacuum concentrator.

Phosphopeptide enrichment was done using 10 mg of TiO_2_ (Zhou *et al*., 2013). Peptides were dissolved in 600 μl of 6% TFA/80% ACN. TiO_2_ was equilibrated with 250 μl of 6% TFA/80% ACN for 15 min and sample binding was done for 30 min. Then TiO_2_ was washed with 3 × 250 μl 6% TFA/80% ACN, 2 × 250 μl 0.5% TFA/50% ACN and 2 × 250 μl 0.1% TFA. Elution of phosphopeptides was done with 100 μl of 10% NH_4_OH and 10 μl 80% ACN/2% FA. Eluates were pooled and dried down in a vacuum concentrator.

Total protein and phosphopeptide samples were measured with LTQ‐Orbitrap Elite (Thermo Fisher Scientific Inc., https://www.thermofisher.com/). Two micrograms of total protein was separated on a PepMap RSLC 75 μm × 50 cm column (Thermo Fisher Scientific) with a 180‐min linear gradient from 2 to 40% of mobile phase B (mobile phase A, 0.1% FA in H_2_O; mobile phase B, 0.1% FA in 90% ACN) with a flow rate of 300 nl min^−1^. Precursor masses in the range 350–1800 Th were measured in the Orbitrap mass analyzer: profile mode, resolution 120 000, automated gain control (AGC) target 1 000 000, injection time 200 ms. MS/MS analysis was done in the linear ion trap for the 20 most intense precursor ions: collision‐induced dissociation fragmentation, normalized collision energy of 35, rapid scan rate, centroid mode, AGC target 5000 and injection time 50 ms. Prediction of ion injection time was used and dynamic exclusion was enabled with a repeat duration of 30 sec, an exclusion list size of 500 and an exclusion duration of 60 sec. Phosphopeptides were dissolved in 11 μl of 1.8% ACN/0.5% FA and 5 μl was loaded on the column. The LC‐MS settings were the same as in the analysis of total protein samples, with a few modifications. The LC‐gradient was 150 min from 2 to 40% of mobile phase B and multistage activation was enabled with neural losses of 24.49, 32.66, 48.999, 97.97, 195.94 and 293.91 Da for the 10 most intense precursor ions.

### Data analysis and statistics

Peptide identification, phosphorylation site mapping and protein and phosphopeptide quantification were performed with MaxQuant 1.4 (http://www.maxquant.org) (Cox and Mann, 2008) and the Andromeda search algorithm (Cox *et al*., 2011) against the TAIR10 protein database. A maximum of two missed cleavages and methionine oxidation and protein N‐terminal acetylation as dynamic modifications were allowed. Mass tolerance was set to 5 p.p.m. for precursor masses and 0.8 Da for the fragment masses. The maximum false discovery rate was set to 1% for both peptide and protein levels and label‐free quantification of protein was done with a peptide ratio count ≥2. Phosphopeptide identification was performed applying similar settings to those described for the total protein analysis. But phosphorylation of serine, threonine and tyrosine residues was additionally allowed to occur as dynamic modifications and three missed cleavages were enabled. Quantification was done at the peptide level.

Filtering and further data processing were done with Perseus 1.5 software. The final total proteomics data set was obtained by filtering the data matrix so that proteins that were identified in all five biological replicates in at least one genotype were included. Before PCA, values were log_2_ transformed and missing values were replaced by random numbers drawn from the normal distribution that represents low‐abundance measurements of each sample. Phosphopeptide data were filtered like the total proteomics data, along with filtering concerning phosphorylation site mapping. Only phosphopeptides that passed the class I criteria (phosphosite probability >75% and score difference >5) (Olsen *et al*., 2006) were included in the final data set. Moreover, phosphopeptide abundance was normalized to the median of each sample, log_2_ transformed and missing values replaced using the same method as in total proteomics data. For presenting means and standard deviations, log_2_‐transformed data were back transformed into the original scale.

Analysis of variance (anova) with a Tukey post‐hoc test was performed with R software. HCA, based on Spearman rank correlation and an average linkage of *z*‐transformed phosphopeptide abundances, was done with Multiexperiment Viewer (MeV) software (Saeed *et al*., 2003). GO terms that were overrepresented in proteomics data were searched with AgriGO (Du *et al*., 2010) and reduced and visualized with REViGO (Supek *et al*., 2011). The following settings were used for AgriGO: background database TAIR10; a hypergeometric test with Yekutieli multi‐test adjustment method was used for statistical testing and the significance level was set to 0.01; ontology type used was plant GO slim. Settings used for REViGO were: medium (0.7) similarity, UniProt Arabidopsis database and SimRel semantic similarity measure. Search of enriched phosphorylation motifs was done with motif‐x (Schwartz and Gygi, 2005; Chou and Schwartz, 2011) with the following settings: >20 occurrences, significance threshold set to 0.000001 and background proteome was the ipi.ARATH database. The presence of sumoylation motifs was searched with Sequence Manipulation Suite Protein Pattern Find (Stothard, 2000). Additionally, SUMO attachment sites and SUMO interaction motifs were predicted with GPS‐SUMO 1.0 (Zhao *et al*., 2014).

## Conflict of Interest

The authors declare no conflict of interest.

## Supporting information


**Figure S1.** Relative abundance of phosphorylated small ubiquitin‐related modifier (SUMO) in different genotypes.

**Figure S2.** Graphic display of up‐ and downregulated proteins with complete annotation of significantly enriched Gene Ontology categories.

**Table S1.** Proteins of Arabidopsis detected and quantified in this work and their abundance changes.

**Table S2.** Phosphoproteins discussed in this work and their abundance changes.

**Table S3.** Matrix of loadings for principal components analysis of total protein abundances.

**Table S4.** Gene Ontology annotation of proteins differentially expressed in *pial1 pial2*,* siz1* or *pial1 pial2 siz1* mutants compared with the Col‐0 wild type.

**Table S5.** Matrix of loadings for principal components analysis of significantly changed phosphopeptides.

**Table S6.** Motifs for small ubiquitin‐related modifier (SUMO) attachment and SUMO binding predicted by program GPS‐SUMO 1.0 in proteins of Table 1.

**Appendix S1.** Supporting Results and Discussion.
