# Supplementary material for: Protein sumoylation and phosphorylation intersect in Arabidopsis signaling
Source: Plant J. 2017 Jun 4;91(3):505–17. doi: 10.1111/tpj.13575 (PMC5518230; doi:10.1111/tpj.13575)
Supplement: Supplementary file 1 — Figure S1. Relative abundance of phosphorylated small ubiquitin‐related modifier (SUMO) in different genotypes. [file TPJ-91-505-s001.pdf]

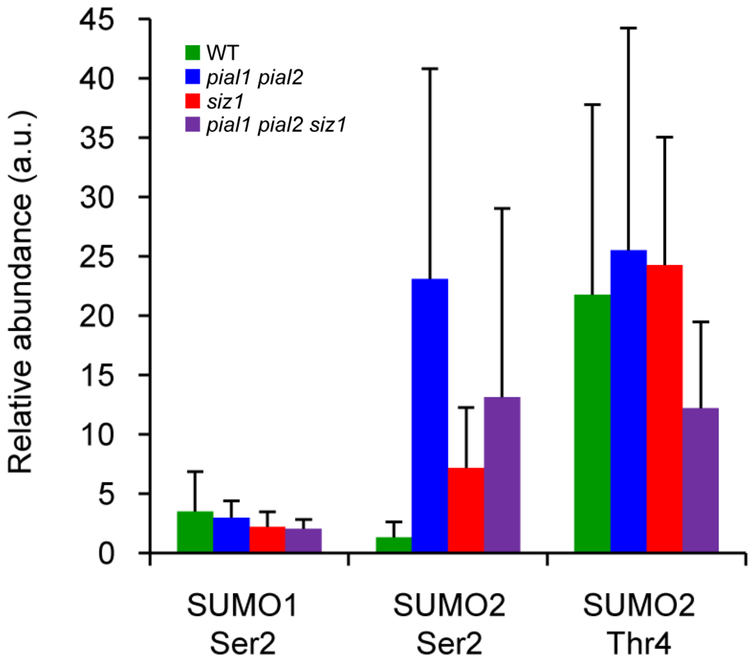

Figure S1. Relative abundance of SUMO1 and SUMO2 phosphorylation on Ser^2 and Thr^4. Values presented are means of 5 biological replicates +/- standard deviation.
