# Supplementary material for: Protein sumoylation and phosphorylation intersect in Arabidopsis signaling
Source: Plant J. 2017 Jun 4;91(3):505–17. doi: 10.1111/tpj.13575 (PMC5518230; doi:10.1111/tpj.13575)
Supplement: Supplementary file 2 — Figure S2. Graphic display of up‐ and downregulated proteins with complete annotation of significantly enriched Gene Ontology categories. [file TPJ-91-505-s002.pdf]

(c)

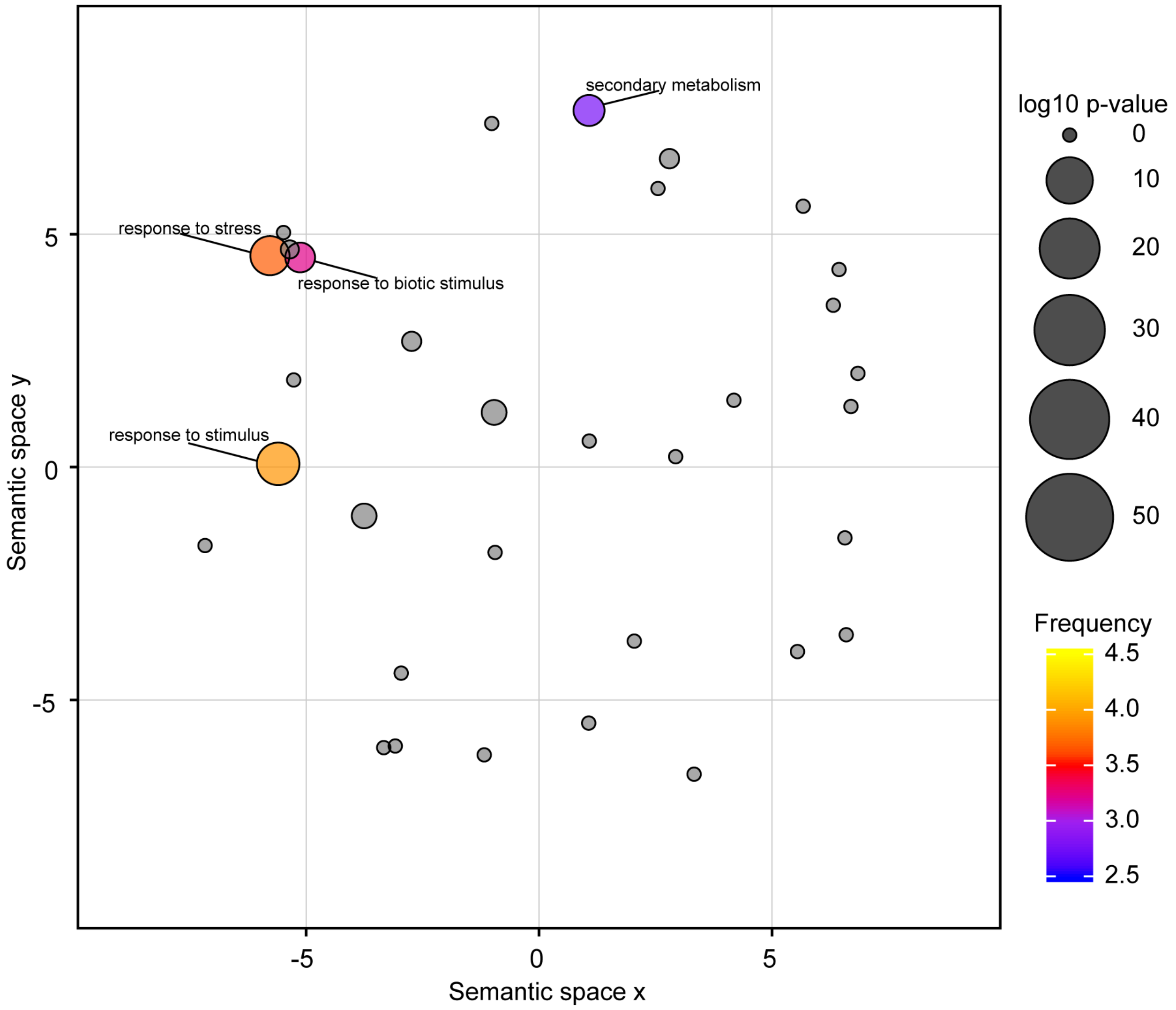

secondary metabolism
response to stress
response to biotic stimulus
response to stimulus
Semantic space y
Semantic space x
5
0
-5
-5
0
5
log10 p-value
0
10
20
30
40
50
Frequency
4.5
4.0
3.5
3.0
2.5

(d)

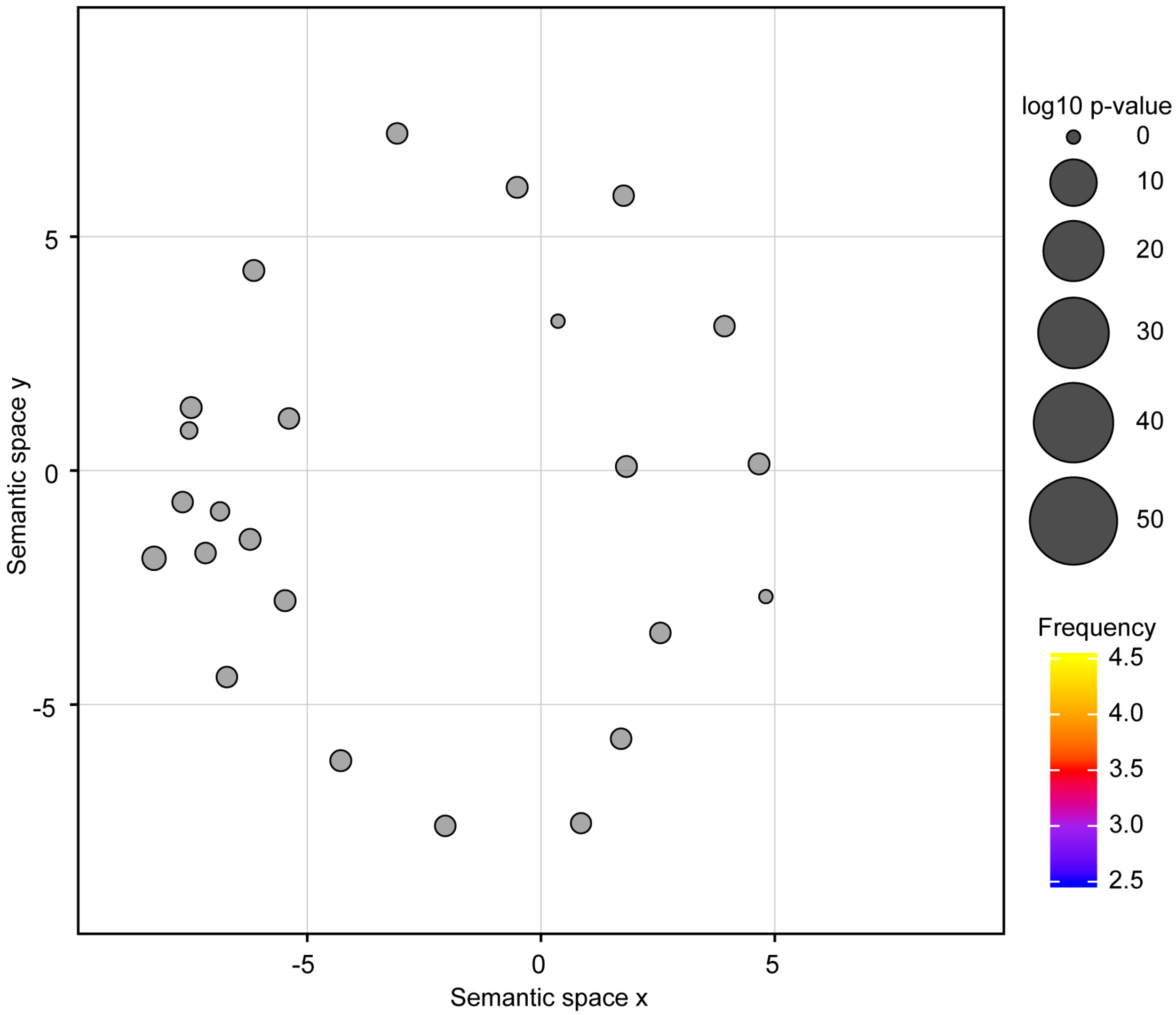

Semantic space y
Semantic space x
5
0
-5
-5
0
5
log10 p-value
0
10
20
30
40
50
Frequency
4.5
4.0
3.5
3.0
2.5

(e)

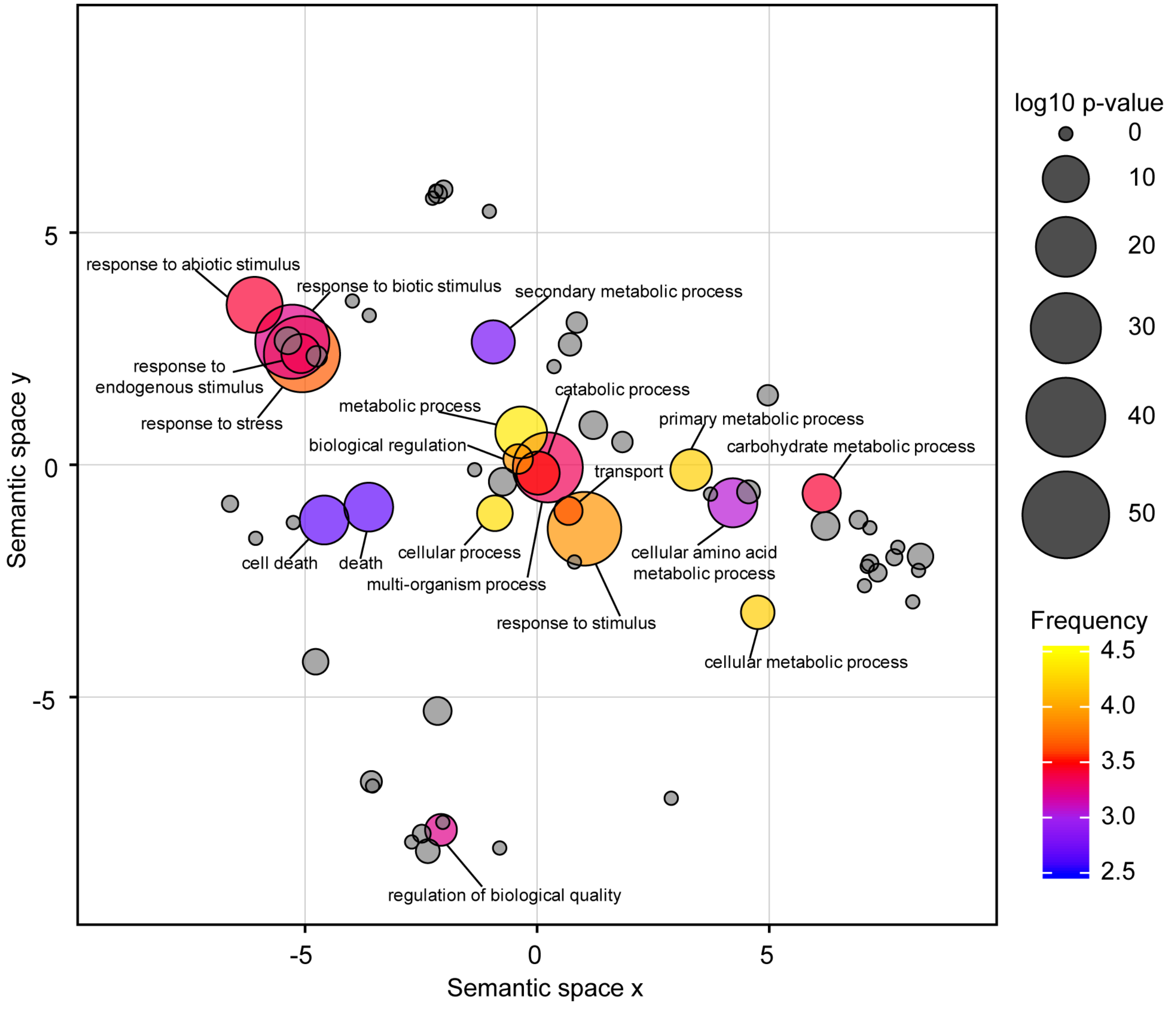

log10 p-value
0
10
20
30
40
50
Frequency
4.5
4.0
3.5
3.0
2.5
Semantic space y
Semantic space x
5
0
-5
response to abiotic stimulus
response to biotic stimulus
secondary metabolic process
response to endogenous stimulus
response to stress
metabolic process
catabolic process
primary metabolic process
carbohydrate metabolic process
biological regulation
transport
cell death
death
cellular process
multi-organism process
cellular amino acid metabolic process
response to stimulus
cellular metabolic process
regulation of biological quality

(f)

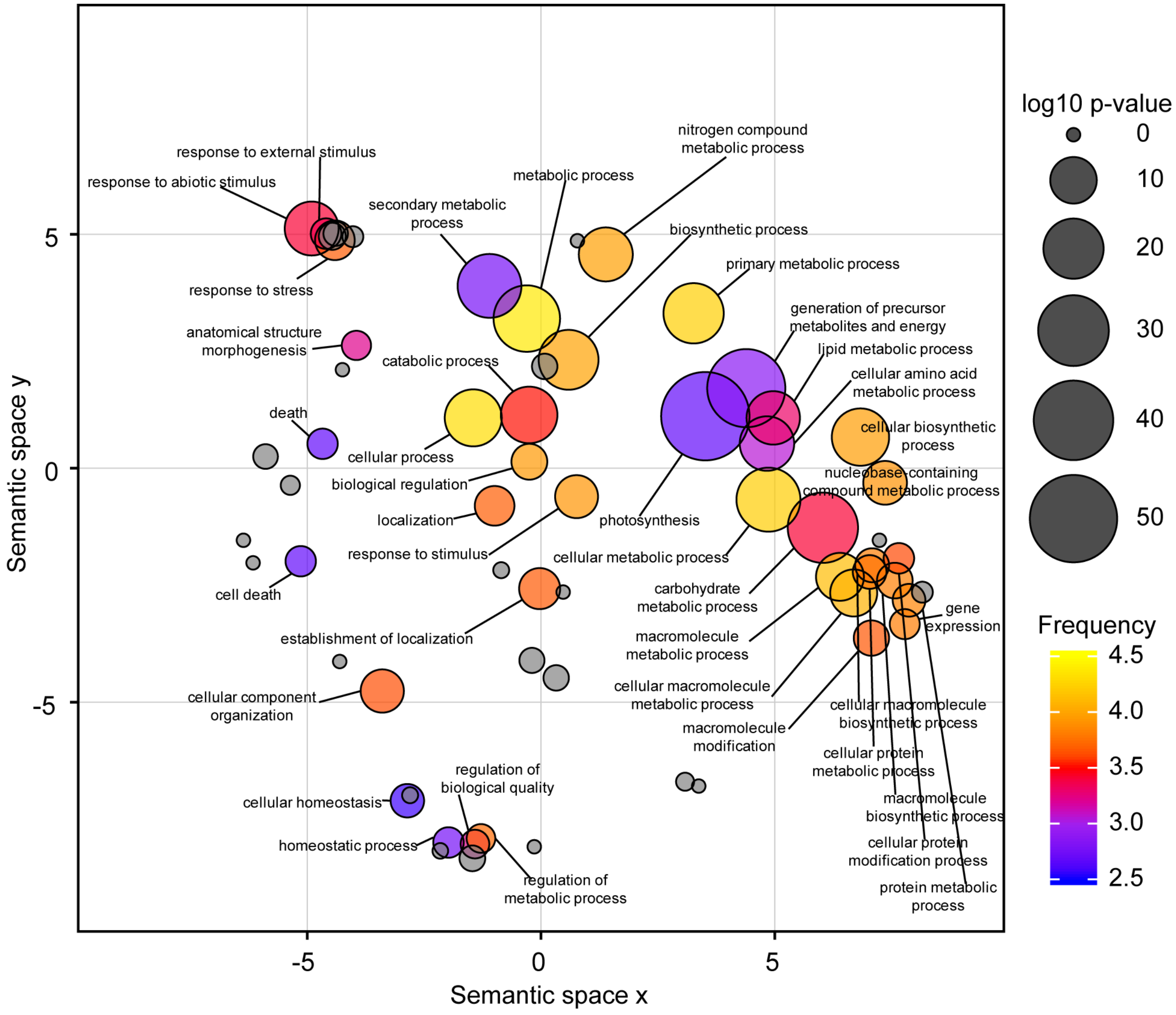

log10 p-value
0
10
20
30
40
50
Frequency
4.5
4.0
3.5
3.0
2.5
Semantic space x
Semantic space y
-5
0
5
response to external stimulus
response to abiotic stimulus
response to stress
secondary metabolic process
metabolic process
nitrogen compound metabolic process
biosynthetic process
primary metabolic process
anatomical structure morphogenesis
catabolic process
generation of precursor metabolites and energy
lipid metabolic process
cellular amino acid metabolic process
death
cellular process
cellular biosynthetic process
biological regulation
nucleobase-containing compound metabolic process
localization
photosynthesis
response to stimulus
cellular metabolic process
cell death
carbohydrate metabolic process
gene expression
establishment of localization
macromolecule metabolic process
cellular component organization
cellular macromolecule metabolic process
cellular macromolecule biosynthetic process
macromolecule modification
cellular protein metabolic process
regulation of biological quality
cellular homeostasis
macromolecule biosynthetic process
homeostatic process
cellular protein modification process
regulation of metabolic process
protein metabolic process

(g)

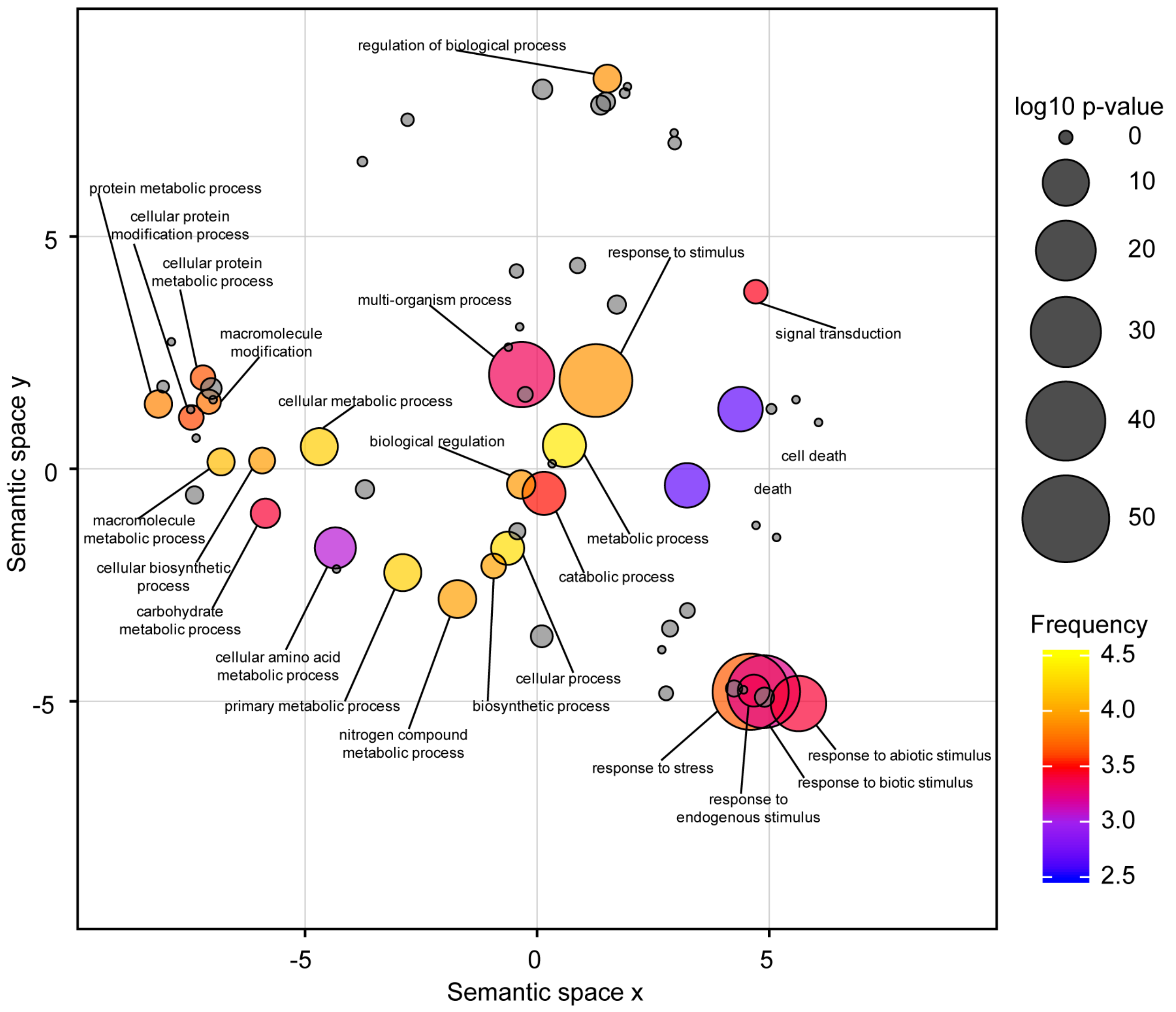
regulation of biological process
protein metabolic process
cellular protein modification process
cellular protein metabolic process
macromolecule modification
response to stimulus
multi-organism process
signal transduction
cellular metabolic process
biological regulation
cell death
death
macromolecule metabolic process
cellular biosynthetic process
carbohydrate metabolic process
metabolic process
catabolic process
cellular amino acid metabolic process
primary metabolic process
cellular process
biosynthetic process
nitrogen compound metabolic process
response to stress
response to abiotic stimulus
response to biotic stimulus
response to endogenous stimulus
Semantic space x
Semantic space y
-5
0
5
-5
0
5
log10 p-value
0
10
20
30
40
50
Frequency
4.5
4.0
3.5
3.0
2.5

(h)

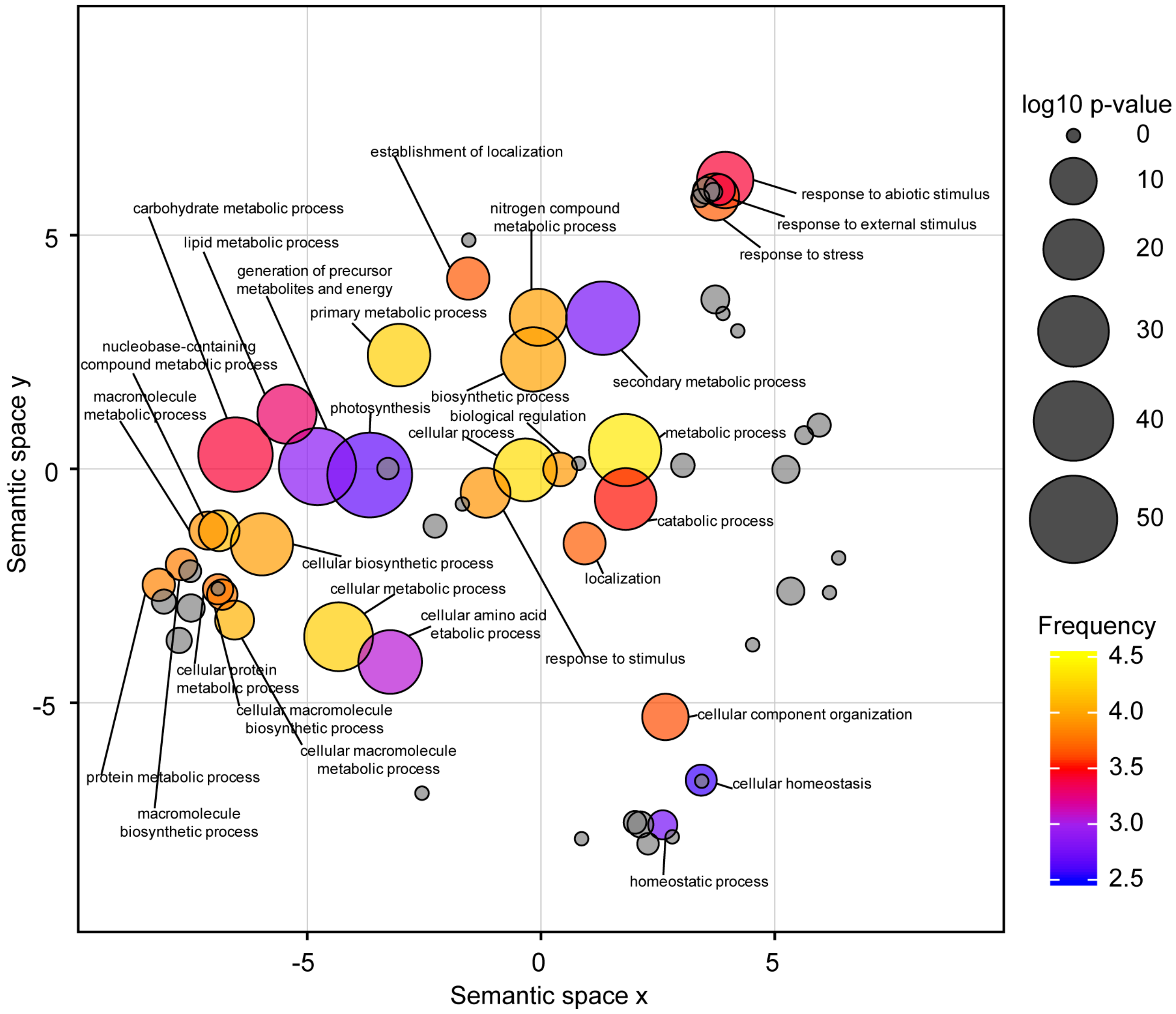

Figure S2. Graphic display of up- and downregulated proteins with complete annotation of significantly enriched GO categories.

Panels **(c)** to **(h)** are identical to those of Figure 3, but contain the names of all significantly enriched GO terms. For individual proteins, see Table S4.
