# Supplementary material for: Protein sumoylation and phosphorylation intersect in Arabidopsis signaling
Source: Plant J. 2017 Jun 4;91(3):505–17. doi: 10.1111/tpj.13575 (PMC5518230; doi:10.1111/tpj.13575)
Supplement: Supplementary file 9 — Appendix S1. Supporting Results and Discussion. [file TPJ-91-505-s009.docx]

**SUPPORTING RESULTS AND DISCUSSION**

**Altered protein abundance in components of plant defense**

NSP5 (AT5G48180) was upregulated in the *pial1 pial2* and in the *pial1 pial2 siz1* mutant and nitrilase 2 (NIT2; AT3G44300) in the triple mutant and in *siz1* plants. Several proteins that were also found to be constitutively induced at mRNA level in the *siz1-2* mutant (Lee et al., 2006) were upregulated in the *siz1* and *pial1 pial2 siz1* mutants. Pathogenesis-related proteins 1 and 5 (PR1; AT2G14610 and PR5; AT1G75040) were upregulated in the mutants *siz1* and *pial1 pial2 siz1*, as were beta-1,3-glucanase 2 (BG2 or PR2; AT3G57260), beta-1,3-glucanase 3 (BG3; AT3G57240) and PR5-like protein pathogenesis-related thaumatin-like protein (CBP1; AT2G28790). Moreover, we detected that putative endochitinase (CHI; AT2G43570), homolog of carrot EP3-3 chitinase (EP3; AT1G74710), two putative chitinases (AT1G02360 and AT4G01700) and a glycosyl hydrolase family protein that has a chitinase insertion domain (AT4G19810) were upregulated in lines that have a mutation in SIZ1. Also, glycine-rich protein 3 short isoform (GRP3S; AT2G05380) was upregulated in *siz1* mutants, consistent with its upregulation by salicylic acid treatment (Park et al., 2001), or when Arabidopsis was inoculated with P. fluorescens (Wang et al., 2005). Even though SIZ1-mediated signalling seemed to have a stronger effect on the regulation of these pathogenesis- and SA-induced proteins, the two other SUMO ligases PIAL1 and PIAL2 were also affecting their expression.

Moreover, glutathione-S-transferases were upregulated in *siz1* and *pial1 pial2 siz1* plants. They are part of several cellular processes, including stress response. Similarly, several peroxidases were upregulated in the *siz1*, *pial1 pial2* and *pial1 pial2 siz1* mutants. Several proteins related to flavonoid and anthocyanin biosynthesis, chalcone synthase (CHS or TT4; AT5G13930), flavanone 3-hydroxylase (F3H or TT6; AT3G51240), flavonol synthase 1 (FLS1; AT5G08640), chalcone isomerase 1 (CHI1 or TT5; AT3G55120) and UDP-glycosyltransferase 78D1 (UGT78D1; AT1G30530), were more abundant in the *siz1* mutant than in other lines including the triple mutant, which displayed levels similar to wild type. Consistent with this, expression of anthocyanin biosynthesis genes was reported to be higher in *siz1-3* mutants, but did not increase in drought as had been found in wild type (Catala et al., 2007).

The increased presence of SA in *siz1* and in triple mutants is predicted to down-regulate genes with JA-inducible character. Some proteins predicted to be JA-inducible showed lower abundance in these mutants. For instance, expression of JA biosynthesis gene LOX2 (AT3G45140) in mutant lines was significantly different from wild type and from each other, while the abundance of AOS (AT5G42650), AOC1 (AT3G25760) and AOC2 (AT3G25770) seemed to depend on the presence of SIZ1. Also, oxophytodienoate-reductase 3 (OPR3; AT2G06050) changed significantly in *siz1* and *pial1 pial2 siz1* mutants, but the decrease compared to wild type was less than 50%. Its expression had a pattern similar to LOX2.

**Changes in the photosynthetic apparatus and in chloroplast proteins**

Chloroplastic proteins were found to be downregulated in *siz1* and *pial1 pial2 siz1* mutants. This could be a consequence of the high SA levels, which direct plants from growth-related processes to defense, but this could also reflect an effect of sumoylation on choloroplast – nucleus communication. The downregulated proteins encompass several subunits of photosystem II (PSII), photosystem I (PSI), as well as proteins from Calvin cycle and photorespiration. Proteins involved in starch synthesis and metabolism were downregulated in *siz1* and *pial1 pial2 siz1* mutants, and the respective GO terms (carbohydrate metabolism) were enriched in GO analysis (Fig. 3F and 3H). Several proteins that have been shown by mutant analyses to have a crucial role in the starch synthesis (reviewed in Streb and Zeeman, 2012) were less abundant in *siz1* and *pial1 pial2 siz1* mutants. Phosphoglucose isomerase 1 (PGI1; AT4G24620), phosphoglucomutases 1, 2 and 3 (PGM1; AT5G51820, PGM2; AT1G70730, PGM3; AT1G23190), ADP glucose pyrophosphorylases 1 and 2 (ADG1 or APS1; AT5G48300, ADG2 or APL2; AT5G19220), granule-bound starch synthase 1 (GBSS1; AT1G32900), and starch synthase 1 (SS1: AT5G24300) were less abundant in *siz1* and *pial1 pial2 siz1* mutants and ADG1 and ADG2 also in the *pial1 pial2* mutant compared to wild type. SIZ1 deficiency leads also to less-starch phenotype (Castro et al., 2015). Furthermore, also starch degrading proteins were less abundant in mutants lacking SIZ1. We found phosphoglucan water dikinase (PWD or AtGWD3; At5g26570), alpha-amylase 3 (AMY3; AT1G69830), beta-amylase 3 (BAM3 or BMY8; AT4G17090), and isoamylase 3 (ISA3; AT4G09020) to be less abundant in the *siz1* and *pial1 pial2 siz1* mutants, and BAM3 also in the *pial1 pial2* mutant. Also, gene expression of BAM3 was downregulated in the *siz1* mutant (Catala et al., 2007). Interestingly, alpha-amylase 1 (AMY1; AT4G25000) protein expression was increased more than four-fold in the mutants lacking SIZ1. This enzyme is secreted to the apoplast and its expression is induced by biotic and abiotic stresses (Doyle et al., 2007). AMY1 has amylase activity in vitro, but is not required for starch breakdown.

**Comparison to transcript abundance data of Han et al (2016)**

We compared our protein abundances to the recently published transcript abundance data for *pial1 pial2* mutants of Han et al. (2016), who studied a novel role of PIAL1 and PIAL2 in transcriptional silencing. Although the growth conditions (and thus protein expression profiles) in the two experiments might not be exactly the same, the data provide hints whether differences in protein abundance are based on transcriptional, or posttranscriptional processes. Interestingly, there was only a small overlap between the transcript dataset of Han et al. (2016) and the protein abundance data of this work. The transcript levels of flavin-dependent oxidoreductase FOX1 (AT1G26380), pathogenesis-related protein 1 (PR1; AT2G14610), patatin-like protein 2 (PLA2A; AT2G26560), P-loop containing nucleoside triphosphate hydrolases superfamily protein (RPL18A; AT4G04180), and ribosomal protein L18e/L15 superfamily protein (AT5G27850) were upregulated in the *pial1 pial2* mutant, but this upregulation on the transcript level did not coincide with altered protein abundance under our growth conditions. However, all these proteins were upregulated at the protein level in *pial1 pial2 siz1* triple mutants. Similarly, Han et al. (2016) found AFL1 (AT3G28270) to be transcriptionally downregulated in *pial1 pial2*. In our study, lack of PIALs increased the AFL1 protein level, but in the *pial1 pial2 siz1* triple mutants, the protein had decreased abundance (Table S1).

**Phosphoproteome analysis**

In addition to principal component analysis of phosphopeptides (Fig. 4), changes in the detected phosphoproteome were subjected to herarchical cluster analysis (Fig. 5). When PCA and HCA results were compared, it turned out that phosphopeptides with highest loading values in PC1 were clustering in the HCA in subclusters 1A and 1C, meaning upregulation in *pial1 pial2 siz1*. Phosphopeptides with the lowest loading values in PC1 clustered in cluster 2 (higher abundance in *siz1*, lowered abundance in the triple mutant) and subcluster 3D (lowered phosphorylation both in *siz1* and in the triple mutant), meaning downregulation *in pial1 pial2 siz1*. High loading values in PC2 are scattered in all subclusters, but enriched in subcluster 1B and cluster 2, meaning upregulation in *siz1*. Phosphopeptides with low loading values in PC2 populated mainly subclusters 1A (lower abundance in *pial1 pial2*, higher abundance in the triple mutant) and 3D. Phosphopeptides with high loading values in PC3 did not clearly cluster in one of the HCA subclusters, but low loadings from PC3 clustered in subcluster 1A and cluster 2.

The obtained clusters differ in the following way. The first cluster contains phosphopeptides that were highly abundant in the *pial1 pial2 siz1* mutant. This cluster was further divided into 3 subclusters. In subcluster 1A are phosphopeptides that are decreased in *pial1 pial2* and increased in the *pial1 pial2 siz1* mutant. Phosphopeptides in this cluster were also less abundant in *siz1* than in the triple mutant, meaning that the lack of all studied SUMO ligases led to higher phosphorylation of these proteins. Phosphopeptides in subcluster 1B had low abundance in wild type and in *pial1 pial2*, and high abundance in lines containing the *siz1* mutation. Subcluster 1C also contains peptides that were more abundantly phosphorylated in the triple mutant. In the second main cluster are phosphopeptides that were differently phosphorylated in *siz1* and *pial1 pial2 siz1*, with lower abundance in *pial1 pial2 siz1* than in *siz1*. The third main cluster was divided in two subclusters. The *siz1* mutation caused lower phosphorylation status of peptides in subcluster 3D. Subcluster 3E contains phosphopeptides which were more abundant in the triple mutant than in *siz1*, and had similar abundance in the *pial1 pial2* and the triple mutant.

**Phosphorylation of proteins regulating RNA transcription**

Clusters 1 and 3 contained several proteins that are involved in RNA metabolism and regulation of transcription. In cluster 1, there were four zinc finger CCCH-type proteins involved in transcription (AT5G12850, AT2G41900, AT3G62330, AT3G51950, and AT5G47430). The total protein level was quantified for AT2G41900, and this was similar in all genotypes (Table S1).

**Connection to hormone signalling and senescence**

In subcluster 1C, there were four proteins that respond to auxin. These are annotated as aluminum-induced proteins (AT3G22850, AT3G15450, AT5G43830, and AT4G27450), all phosphorylated at the “same” serine (Ser215-219). These proteins were more phosphorylated in all mutants than in the wild type, but also their protein abundance was higher, probably meaning that the protein synthesis or turnover, rather than the phosphorylation efficiency, was affected. AT3G22850 and AT3G15450 (SEN5) are involved in energy metabolism (Zou et al., 2009; Rodrigues et al., 2013). SEN5 is a SnRK1 target gene and its expression is induced by ABA and darkness. Aluminum- and auxin-induced protein AT4G27450 was upregulated in the *pial1 pial2 siz1* mutant, but not in *siz1* or in *pial1 pial2*. AT5G43830 is also ethylene-regulated, and its phosphorylation is enhanced by ethylene treatment, whereas no ethylene dependence was reported for two other aluminum-induced proteins (AT3G22850 and AT4G27450) (Li et al., 2009), which were upregulated in our study. This indicates that the induction of proteins and phosphorylation abundance in *pial1 pial2 siz1* is ethylene-independent.

**Phosphorylation changes of chloroplast proteins in *pial1 pial2 siz1***

As seen in total proteome results, photosynthesis-related proteins were affected in SUMO ligase mutants. In the cluster 1 of Figure 5 are three chloroplast proteins that were more phosphorylated in the *pial1 pial2 siz1* mutant. Plastid transcriptionally active 16 (pTAC16; AT3G46780) is a target for STN7 phosphorylation on Thr451 (Ingelsson and Vener, 2012). In our data set, Thr451 phosphorylation was not affected according to the ANOVA (p<0.05), but phosphorylation of Ser395 was upregulated in the *pial1 pial2 siz1* mutant. The other chloroplast localized protein is thylakoid soluble phosphoprotein (TSP9; AT3G47070). It is also a substrate for STN7 and STN8, and the Thr64/66 phosphorylation detected in our study was reported as STN7/STN8-dependent (Ingelsson and Vener, 2012). TSP9 is a soluble protein and it has been speculated that its unphosphorylated form is associated with the thylakoid membrane. Light-induced phosphorylation causes release from the membrane (Carlberg et al., 2003). Phosphorylation of TSP9 might thus regulate light utilization by facilitating the migration of light-harvesting proteins between the two photosystems (Pesaresi et al., 2011). The increases of pTAC16 and TSP9 were specific for the extent of phosphorylation, since the protein level of pTAC16 was lower in the triple mutant than in wild type, and TPS9 abundance was similar in mutant and wild type (Table S1). Photosystem I reaction center subunit IV A (PSAE1; AT4G28750) is part of the PSI and provides a docking site for soluble ferredoxin on the stromal side of the thylakoid membrane (Amunts and Nelson, 2009). It was also significantly more phosphorylated in the *pial1 pial2 siz1* mutant than in the wild type, even though PSAE1 protein amount was significantly lower in all mutants compared to the wild type (Table S1). Phosphorylation of PSAE1 has been detected previously, but the role of phosphorylation is unknown. On the other hand, chloroplastic ATP synthase subunit beta (ATPB; ATCG00480) was in cluster 3 of Figure 5, and its phosphorylation level was lower in *siz1* mutant than in the wild type and in *pial1 pial2*. This decrease in phosphorylation might, however, reflect the protein abundance rather than phosphorylation efficiency, because the level of ATPB was lower in *pial1 pial2 siz1* and *siz1* plants (Table S1).

**Other protein abundances and alterations in the phosphorylation pattern**

The second major cluster of Figure 5, cluster 2, contains the phosphopeptides downregulated in the *pial1 pial2 siz1* mutant, and includes two LEA proteins that showed increased phosphorylation in *siz1* compared to wild type. Furthermore, cluster 2 contains 10 phosphopeptides that were differentially phosphorylated between *pial1 pial2 siz1* and *siz1*. Six phosphopeptides were significantly more phosphorylated in the *siz1* than in the *pial1 pial2* mutant.

Dynamin proteins DRP2A and DRP2B are the only dynamins in Arabidopsis that contain all the essential domains for their function (Taylor, 2011). They are highly similar and functionally redundant and they are expressed throughout the plant. In the *pial1 pial2 siz1* mutant, the phosphorylation of DRP2A and DRP2B was higher than in wild type, but also their protein abundance was increased (Table S1). Likewise, components of the cytoskeleton were affected. Two profilins (PRF1; AT2G19760 and PRF2; AT4G29350), which are expressed in vegetative tissues (Jeong et al., 2006), were less abundant in plants lacking SIZ1, and three gamma interferon responsive lysosomal thiol (GILT) reductase family proteins (AT4G12890, AT4G12960 and AT4G12870) were less abundant in the triple mutant compared to the *siz1* single mutant.

**SUPPORTING REFERENCES**

Amunts, A., and Nelson, N. (2009). Plant photosystem I design in the light of evolution. Structure 17, 637-650.

Carlberg, I., Hansson, M., Kieselbach, T., Schroder, W.P., Andersson, B., and Vener, A.V. (2003). A novel plant protein undergoing light-induced phosphorylation and release from the photosynthetic thylakoid membranes. Proc Natl Acad Sci U S A 100, 757-762.

Castro, P.H., Verde, N., Lourenco, T., Magalhaes, A.P., Tavares, R.M., Bejarano, E.R., and Azevedo, H. (2015). SIZ1-Dependent Post-Translational Modification by SUMO Modulates Sugar Signaling and Metabolism in Arabidopsis thaliana. Plant & cell physiology 56, 2297-2311.

Catala, R., Ouyang, J., Abreu, I.A., Hu, Y., Seo, H., Zhang, X., and Chua, N.H. (2007). The Arabidopsis E3 SUMO ligase SIZ1 regulates plant growth and drought responses. Plant Cell 19, 2952-2966.

Doyle, E.A., Lane, A.M., Sides, J.M., Mudgett, M.B., and Monroe, J.D. (2007). An alpha-amylase (At4g25000) in Arabidopsis leaves is secreted and induced by biotic and abiotic stress. Plant Cell Environ 30, 388-398.

Ingelsson, B., and Vener, A.V. (2012). Phosphoproteomics of Arabidopsis chloroplasts reveals involvement of the STN7 kinase in phosphorylation of nucleoid protein pTAC16. FEBS Lett 586, 1265-1271.

Jeong, Y.M., Mun, J.H., Lee, I., Woo, J.C., Hong, C.B., and Kim, S.G. (2006). Distinct roles of the first introns on the expression of Arabidopsis profilin gene family members. Plant Physiol 140, 196-209.

Li, H., Wong, W.S., Zhu, L., Guo, H.W., Ecker, J., and Li, N. (2009). Phosphoproteomic analysis of ethylene-regulated protein phosphorylation in etiolated seedlings of Arabidopsis mutant ein2 using two-dimensional separations coupled with a hybrid quadrupole time-of-flight mass spectrometer. Proteomics 9, 1646-1661.

Park, A.R., Cho, S.K., Yun, U.J., Jin, M.Y., Lee, S.H., Sachetto-Martins, G., and Park, O.K. (2001). Interaction of the Arabidopsis receptor protein kinase Wak1 with a glycine-rich protein, AtGRP-3. J Biol Chem 276, 26688-26693.

Pesaresi, P., Pribil, M., Wunder, T., and Leister, D. (2011). Dynamics of reversible protein phosphorylation in thylakoids of flowering plants: the roles of STN7, STN8 and TAP38. Biochimica et biophysica acta 1807, 887-896.

Rodrigues, A., Adamo, M., Crozet, P., Margalha, L., Confraria, A., Martinho, C., Elias, A., Rabissi, A., Lumbreras, V., Gonzalez-Guzman, M., Antoni, R., Rodriguez, P.L., and Baena-Gonzalez, E. (2013). ABI1 and PP2CA phosphatases are negative regulators of Snf1-related protein kinase1 signaling in Arabidopsis. Plant Cell 25, 3871-3884.

Streb, S., and Zeeman, S.C. (2012). Starch metabolism in Arabidopsis. Arabidopsis Book 10, e0160.

Taylor, N.G. (2011). A role for Arabidopsis dynamin related proteins DRP2A/B in endocytosis; DRP2 function is essential for plant growth. Plant Mol Biol 76, 117-129.

Wang, Y., Ohara, Y., Nakayashiki, H., Tosa, Y., and Mayama, S. (2005). Microarray analysis of the gene expression profile induced by the endophytic plant growth-promoting rhizobacteria, Pseudomonas fluorescens FPT9601-T5 in Arabidopsis. Molec plant-microbe interact 18, 385-396.

Zou, J., Song, L., Zhang, W., Wang, Y., Ruan, S., and Wu, W.H. (2009). Comparative proteomic analysis of Arabidopsis mature pollen and germinated pollen. J Integr Plant Biol 51, 438-455.
