# Supplementary material for: Protein sumoylation and phosphorylation intersect in Arabidopsis signaling
Source: Plant J. 2017 Jun 4;91(3):505–17. doi: 10.1111/tpj.13575 (PMC5518230; doi:10.1111/tpj.13575)
Supplement: Supplementary file 10 [file TPJ-91-505-s010.docx]

**SUPPORTING INFORMATION LEGENDS**

**Supporting Figure S1.** Relative abundance of SUMO1 and SUMO2 phosphorylation on Ser^2^

and Thr^4^. Values presented are means of 5 biological replicates +/- standard deviation.

**Supporting Figure S2.** Graphic display of up- and downregulated proteins with complete annotation of significantly enriched GO categories. Panels (**c**) to (**h**) are identical to those of Figure 3, but contain the names of all significantly enriched GO terms. For individual proteins, see Table S4.

**Supporting Table S1.** Proteins of Arabidopsis detected and quantified in this work and their abundance changes.

**Supporting Table S2.** Phosphoproteins discussed in this work and their abundance changes.

**Supporting Table S3.** Matrix of loadings for PCA of total protein abundances.

**Supporting Table S4.** GO annotation of proteins differentially expressed in *pial1 pial2*, *siz1* or *pial1 pial2 siz1* mutants compared to the Col-0 wild type.

**Supporting Table S5.** Matrix of loadings for PCA of significantly changed phosphopeptides.

**Supporting Table S6.** Motifs for SUMO attachment and SUMO binding predicted by program GPS-SUMO 1.0 in proteins of Table 1.

**Appendix S1.** Supporting Results and Discussion.
